# Adaptation of codon usage to tRNA I34 modification controls translation kinetics and proteome landscape

**DOI:** 10.1371/journal.pgen.1008836

**Published:** 2020-06-01

**Authors:** Xueliang Lyu, Qian Yang, Lin Li, Yunkun Dang, Zhipeng Zhou, She Chen, Yi Liu

**Affiliations:** 1 State Key Laboratory of Agricultural Microbiology, College of Plant Science and Technology, Huazhong Agricultural University, Wuhan, Hubei, China; 2 Department of Physiology, The University of Texas Southwestern Medical Center,Harry Hines Blvd., Dallas, Texas, United States of America; 3 National Institute of Biological Sciences, Changping District, Beijing, China; 4 State Key Laboratory for Conservation and Utilization of Bio-Resources and Center for Life Science, School of Life Sciences, Yunnan University, Kunming, China; 5 College of Life Sciences and Technology, Huazhong Agricultural University, Wuhan, Hubei, China; Oregon State University, UNITED STATES

## Abstract

Codon usage bias is a universal feature of all genomes and plays an important role in regulating protein expression levels. Modification of adenosine to inosine at the tRNA anticodon wobble position (I34) by adenosine deaminases (ADATs) is observed in all eukaryotes and has been proposed to explain the correlation between codon usage and tRNA pool. However, how the tRNA pool is affected by I34 modification to influence codon usage-dependent gene expression is unclear. Using *Neurospora crassa* as a model system, by combining molecular, biochemical and bioinformatics analyses, we show that silencing of *adat2* expression severely impaired the I34 modification levels for the ADAT-related tRNAs, resulting in major ADAT-related tRNA profile changes and reprogramming of translation elongation kinetics on ADAT-related codons. *adat2* silencing also caused genome-wide codon usage-biased ribosome pausing on mRNAs and proteome landscape changes, leading to selective translational repression or induction of different mRNAs. The induced expression of CPC-1, the *Neurospora* ortholog of yeast GCN4p, mediates the transcriptional response after *adat2* silencing and amino acid starvation. Together, our results demonstrate that the tRNA I34 modification by ADAT plays a major role in driving codon usage-biased translation to shape proteome landscape.

## Author summary

Modification of transfer RNA (tRNA) can have profound impacts on gene expression by shaping cellular tRNA pool. How codon usage bias and tRNA profiles synergistically regulate gene expression is unclear. By combining molecular, biochemical and bioinformatics analyses, we showed that the correlation between genome codon usage and tRNA I34 (inosine 34) modification modulates translation elongation kinetics and proteome landscape. Inhibition of tRNA I34 modification causes codon usage-dependent ribosome pausing on mRNAs during translation and changes cellular protein contents in a codon usage biased manner. Together, our results demonstrate that the tRNA I34 modification plays a major role in driving codon usage-dependent translation to determine proteome landscape in a eukaryotic organism.

## Introduction

In eukaryotes, more than 50 different chemical modifications at different positions of tRNAs have been described (RNA modification database https://mods.rna.albany.edu/) [1]. These modifications are important for tRNA structure, function, and stability, and some modifications have been shown to impact codon-biased protein expression [2–6]. tRNA modifications in the anticodon loop can have major impacts on tRNA decoding functions. In bacteria and eukaryotes, although not archaea, the wobble base adenosine at the position 34 of several tRNAs can be converted to inosine (A-to-I editing) by tRNA-adenosine deaminases, known as ADATs [7–10]. In most bacteria, tRNA^Arg^(ACG) is the only substrate of homodimeric TadA, although I34 can be also found in several tRNAs other than tRNA^Arg^(ACG) within a few species [11], whereas in most of eukaryotes, seven or eight families of tRNAs (ADAT-related tRNAs: tRNA^Arg^(ACG), tRNA^Ala^(AGC), tRNA^Ile^(AAU), tRNA^Leu^(AAG), tRNA^Pro^(AGG), tRNA^Ser^(AGA), tRNA^Thr^(AGU) and tRNA^Val^(AAC)) are edited by heterodimeric ADATs composed of ADAT2/Tad2 and ADAT3/Tad3 [7,12,13].

Due to the degeneracy of the genetic code, most amino acids are encoded by two to six synonymous codons. Codon usage bias, the preference for certain synonymous codons for almost all amino acids, is a universal feature of all genomes [14–17]. Codon usage bias plays an important role in determining gene expression levels at both translational and transcriptional levels in eukaryotes [18–23]. A positive correlation between codon usage bias and transfer RNA (tRNA) expression is thought to allow genes with more optimal codons to be more efficiently translated than those enriched with rare codons [17,24–30]. Preferred codons enhance translation elongation rate, whereas rare codons causes translation pausing and premature translation termination [31–34]. As a result, codon usage regulates mRNA translation efficiency.

Although many fungal, insect and mammalian organisms exhibit a bias for C at the wobble positions, tRNAs with GNN anticodons are absent from their genomes. Unmodified A34 base pairs with uridine (U) but I34 is able to form wobble pairs with C, U, and A [7–10]. Thus, the INN anticodons allow tRNAs to recognize more codons. A34-to-I editing by ADATs has been proposed to explain the correlation between codon usage and tRNA abundance in both bacteria and eukaryotes [35]. However, because ADAT is an essential enzyme in eukaryotes [7,36], experimental determination of its biological function is difficult. In addition, the extent of A34-to-I editing in eukaryotic tRNAs and how it influences translation and proteome composition in a codon usage-dependent manner *in vivo* are unclear.

The filamentous fungus *Neurospora crassa* exhibits a strong codon usage bias for C/G at the wobble positions and has been used as a model system to understand the functions of codon usage biases [37,38]. Our previous research showed that the presence of common codons speeds up the local rate of translation elongation, whereas rare codons slow down translation elongation rate, and result in ribosome stalling and premature termination in *Neurospora* [31,34]. Thus, codon usage plays an important role in regulating mRNA translation efficiency. In addition, we also showed that codon usage regulates gene transcription efficiency [22,39]. These results led us to propose that codon usage represents an additional layer of the genetic code that determines both protein structure and gene expression level. Of the ADAT-related codon families, NNC codons are the most preferred in *Neurospora* [37,38]. However, like most of the eukaryotic organisms, the corresponding tRNAs with GNN anticodons are absent in the *Neurospora* genome, and the tRNAs with ANN anticodons are the most abundant. These results suggest that the translation of NNC codons depends on the A34-to-I editing at the anticodons of ANN tRNAs by the ADATs.

Previous studies showed that both codon usage and tRNA pool plays an important role in modulating gene expression at the translational level [25,30,40–45]. In addition to the regulation of tRNA at transcriptional level, many tRNA modifications are dynamically regulated [5,46], which can affect tRNA abundance or their decoding ability. A recent study showed that human tRNA I34 modification efficiency is regulated at different developmental stages [47]. These findings suggest that both tRNA modification and codon usage play important roles in regulating gene expression. However, the mechanism of how codon usage and tRNA modification synergistically influence the translation kinetics and proteome are unclear. To explore the underlying mechanism, we created a *Neurospora* strain in which the expression of the *adat2* gene can be inducibly silenced. We found that the silencing of *adat2* expression abolished most tRNA I34 modification and resulted in major ADAT-related tRNA profile changes and reprogramming of translation elongation kinetics on ADAT-related codons. We demonstrated that the changes in the ADAT-related tRNA profile caused codon usage-biased translation kinetics and proteome landscape changes. Together, our results demonstrate that A34-to-I editing of tRNAs play a major role in driving codon usage-biased gene expression and the adaption of codon usage and tRNA pool determines the translation kinetics to shape the proteome in a eukaryotic organism.

## Results

### Co-evolution among tRNA-dependent adenosine deaminases, tRNA composition and codon usage frequency

**Fig 1:**
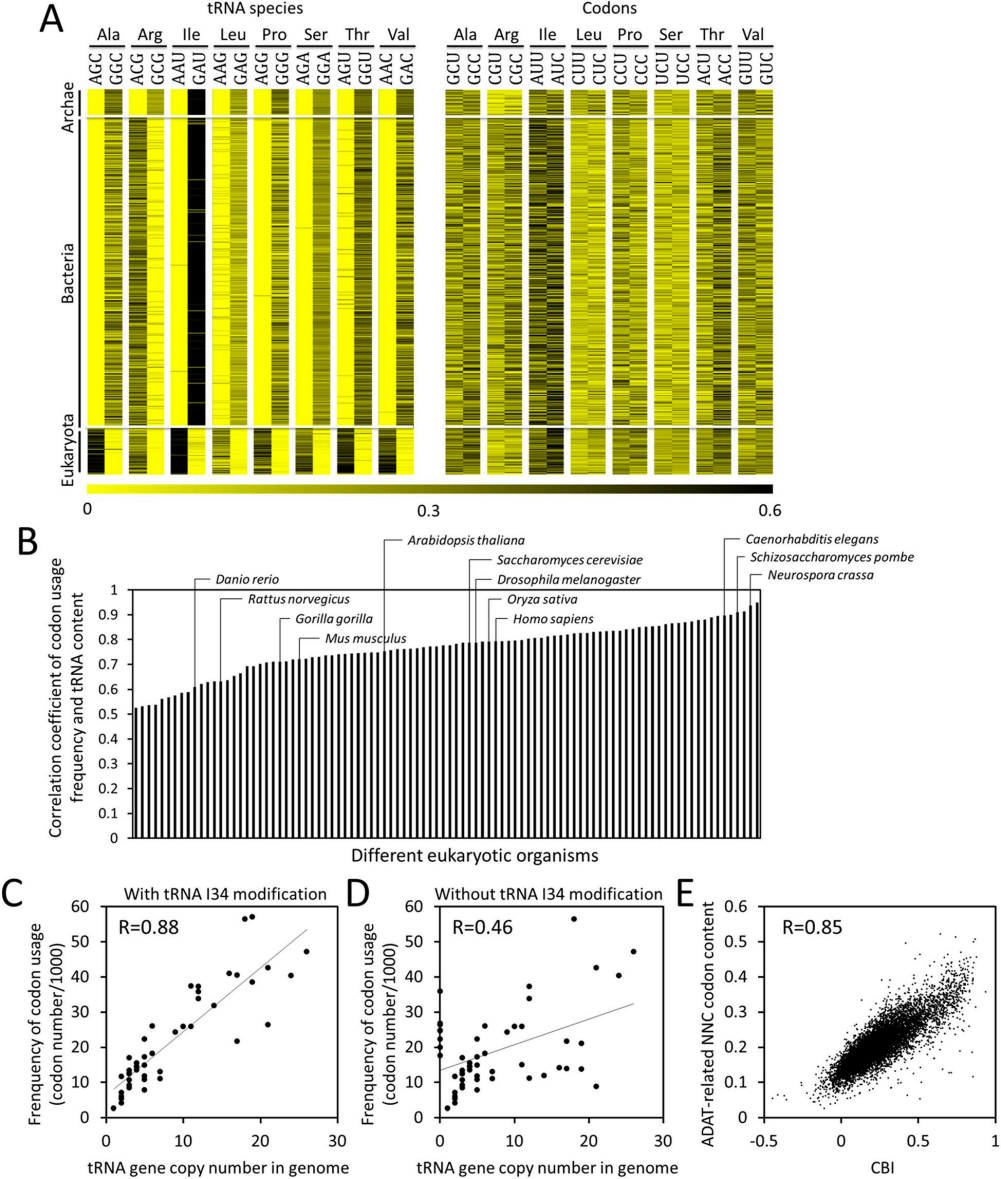
Relationship among tRNA-dependent adenosine deaminases, tRNA composition and codon usage frequency. **(A)** The relative abundance of ADAT-related tRNA species in each tRNA family (left) and the corresponding relative codon usage frequency in each codon family (right) of 959 species from archaea, bacteria, and eukaryotes, respectively. The tRNA type is designated by its anticodon and encoded amino acid. The color code depicts the relative tRNA abundance (based on tRNA gene copy number in genomes) or the relative codon usage frequency in each family. **(B)** Histogram showing the Pearson’s correlation coefficient between tRNA content and genome-wide codon usage frequency in 96 eukaryotic organisms when I:C, I:U and G:U wobble events are taken into account. Major model organisms are indicated. tRNA content was calculated as the gene copy number of a specific tRNA gene divided by total tRNA gene number in each genome. **(C)** Scatter plot showing the correlation between tRNA gene copy number and codon usage frequency in *N*. *crassa* genome when I:C, I:U and G:U wobble events are taken into consideration. **(D)** Scatter plot showing the correlation between tRNA gene copy number and codon usage frequency in the *N*. *crassa* genome when tRNA I34 modification (I:C, I:U wobble events) was not considered. **(E)** Scatter plot showing the strong correlation between gene codon optimality measured by CBI values and ADAT-related NNC codon contents in *N*. *crassa*. R values in C, D and E represent respective Pearson’s correlation coefficient.

ADATs are found in bacterial genomes and are ubiquitous in eukaryotic organisms. To analyze the relationship between the ADAT-related tRNA composition and genome-wide codon usage frequency, we determined the relative tRNA abundances in the eight ADAT-related tRNA families and their corresponding relative codon usage frequencies in 959 archaeal, bacterial, and eukaryotic organisms (Fig 1A). In archaeal genomes, there are no tRNAs with ANN anticodons, and the tRNAs with GNN anticodons are dominant; thus, decoding of NNU codons depends on tRNAs that can form G:U wobble at the third position of codon-anticodon interaction. In most of bacteria, the TadA modifies the anticodon of tRNA^Arg^ from ACG to ICG but cannot modify other tRNAs. As a result, tRNA^Arg^(ACG) is the dominant tRNA^Arg^ species and almost all tRNA^Arg^(GCG) species are missing from bacterial genomes. In contrast, seven other tRNA(GNN) species are present in bacterial genomes. In the vast majority of eukaryotes, the heterodimeric ADATs can modify seven to eight different tRNA species with ANN anticodons (tRNA^Arg^(ACG), tRNA^Ala^(AGC), tRNA^Ile^(AAU), tRNA^Leu^(AAG), tRNA^Pro^(AGG), tRNA^Ser^(AGA), tRNA^Thr^(AGU) and tRNA^Val^(AAC)). As expected, these tRNA(ANNs) are the dominant tRNA species in the eight tRNA families in eukaryotic organisms. As with the tRNA^Arg^(GCG) in bacteria, almost all the tRNA(GNN) species within these families became extinct in eukaryotes during evolution (Fig 1A). These results, which are consistent with previous studies [35], suggest that the tRNA composition might be shaped by ADAT-associated tRNA modification, and that the tRNAs with INN anticodons are strongly selected over tRNAs with GNN anticodons in Bacteria and Eukarya. On the other hand, despite the disappearance of tRNAs with GNN anticodons, NNC codons are the preferred codons in the majority of eukaryotic organisms examined (Fig 1A), suggesting that the I:C base pairing at the third position of codon-anticodon interaction allows tRNA(INN) species to efficiently decode NNC codons.

When tRNA I34 modification is considered, strong positive correlations between the tRNA contents and genome-wide codon usage frequencies were seen in 96 eukaryotic organisms (Fig 1B), indicating that although the codon usage bias is different in various organisms, the tRNA pool and codon usage frequency strongly correlate in their genomes. In *Neurospora*, the Pearson’s correlation coefficient between the tRNA gene copy number and codon usage frequency is 0.88 (Fig 1C). However, when tRNA I34 modification is not considered in the analysis, the Pearson’s correlation coefficient dropped to 0.46 (Fig 1D). Consistent with the previous study [35], these results indicate that tRNA I34 modification plays an important role in determining the strong positive correlations between tRNA pools and codon usage frequencies.

To determine the role of ADAT-related NNC codons in determining the overall codon optimality of a gene, we examined the correlation between the ADAT-related NNC codon contents and gene codon bias index (CBI). CBI of 1 indicates that a gene has extreme codon bias, and a CBI value of 0 indicates completely random codon usage [19,48]. As shown in Fig 1E, there is a high correlation between the ADAT-related NNC codon content and CBI, suggesting that the ADAT-related NNC codon contents play a major role in determining gene codon optimality in *Neurospora*.

### ADAT2 is required for tRNA adenosine deamination in *Neurospora*

**Fig 2:**
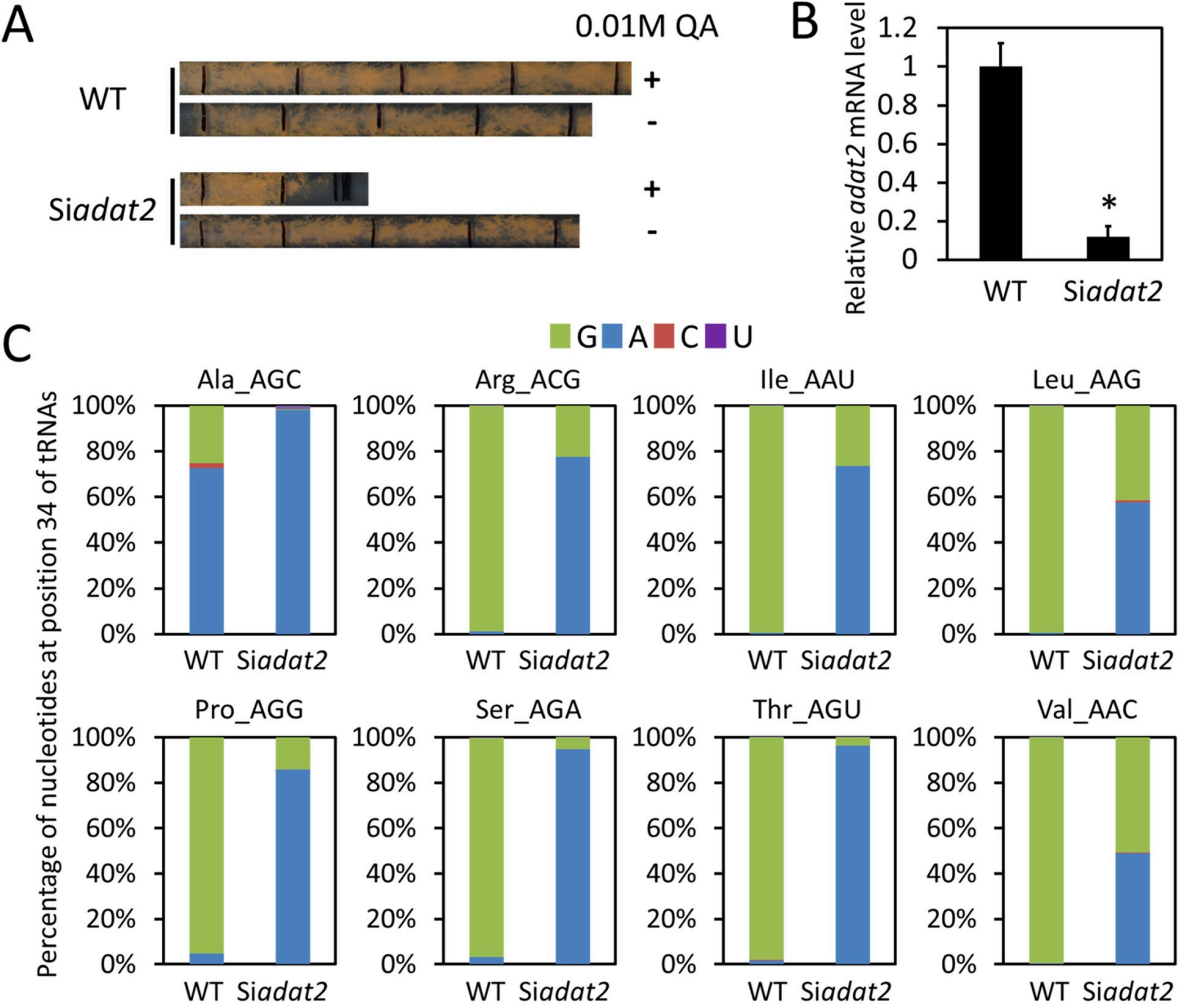
ADAT2 is required for the I34 modification of eight ADAT-related tRNA species. **(A)** Race tube assay comparing the growth phenotypes of the WT and Si*adat2* strains with/without QA. Each black line on the race tube marks the growth front every 24 hrs. **(B)** The relative *adat2* mRNA levels detected by quantitative reverse transcription (qRT)-PCR in the WT and Si*adat2* strains. The expression levels of *adat2* were normalized to that of *β-tubulin*. The *adat2* expression level in the WT strain was set as 1.0. Data are means ± SD. *, P < 0.05, as determined by Student's two-tailed t-test. **(C)** Bar charts showing the proportions of G, A, C, and U contents detected by tRNA sequencing at the position 34 of each tRNA in the WT and Si*adat2* strains. Inosine is read as G by sequencing. The tRNA species with anticodons are indicated at the top of each bar chart.

*adat2* (NCU01888) is predicted to encode the catalytic subunit of heterodimeric ADAT in *Neurospora*. A homokaryotic *adat2* deletion strain could not be obtained in *Neurospora*, consistent with ADAT as an essential enzyme in eukaryotes. To experimentally confirm the function of ADAT in tRNA A34 deamination and its role in shaping tRNA profile, we created a *Neurospora* strain (Si*adat2*), in which the expression of *adat2* can be inducibly silenced by expressing an *adat2*-specific inverted repeat which results in the formation of a long 0.8 kb double-stranded RNA (dsRNA) [49]. The *adat2* dsRNA construct is under the control of a quinic acid (QA) inducible promoter. The production of a long dsRNA in cells generates numerous distinct but gene-specific siRNA species and each species presents at a very low concentration. Although each species of dsRNA-derived siRNA contributes to silencing, each species alone is not sufficient to cause silencing due to its low concentration. As a result, dsRNA results in efficient gene silencing with minimal off-target effect. The Si*adat2* strain exhibited normal growth in the absence of the QA inducer. In the presence of QA, however, cell growth was dramatically impaired after one day of silencing, consistent with the essential role of *adat2* in *Neurospora* survival (Fig 2A). As expected, the silencing of *adat2* led to a dramatic *adat2* mRNA level decrease (Fig 2B).

To examine the impact of *adat2* silencing on tRNA A34 deamination, we extracted tRNAs from the WT and Si*adat2* strains grown in the presence of QA and performed high-throughput tRNA sequencing. If A34 in tRNAs is deaminated to become I34, the “I” will be read as “G” by cDNA sequencing. To avoid potential issues of tRNA sequencing, we only focused on determining the percentage of tRNAs with/without the A34-to-I editing for the eight ADAT-related tRNAs. In the WT strain, all but one of the ADAT-related tRNA species were very highly modified at the position 34 (Fig 2C). The proportions of G (indicating A-to-I editing) at the A34 position for seven ADAT-related tRNAs were near 100% (Fig 2C). The low tRNA^Ala^(AGC), A34 to I modification may be due to low sequencing coverage of this specific tRNA species in this experiment as A34 to I modification for tRNA^Ala^(AGC) was near 100% in additional tRNA sequencing of the wild-type (WT) strain under different conditions (S1A Fig). In the Si*adat2* strain, however, the A34 deamination efficiencies were all dramatically decreased. A34 deamination was almost completely abolished in three tRNAs (tRNA^Ala^(AGC), tRNA^Ser^(AGA), and tRNA^Thr^(AGU) and was reduced to 15–50% for the other five tRNAs (Fig 2C). The small percentages of reads containing C34 for some of tRNAs were likely caused by sequencing errors. These results indicate that ADAT2 is required for tRNA adenosine deamination at A34. Thus *adat2* silencing resulted in major changes in the ADAT-related tRNA profile in *Neurospora*: the relative amounts of tRNAs with INN anticodons were dramatically decreased, while tRNAs with ANN anticodons became more abundant than the tRNAs with INN anticodons in these tRNA families.

To rule out the possibility that the decreased A34 deamination was caused by the indirect effect of amino acid starvation condition, we performed tRNA-seq in the WT, *siadat2* strains and the WT strain treated with 3-aminotriazole (3-AT), which results in amino acid starvation in *Neurospora* [50,51]. As shown in S1B Fig, in contrast to *adat2* silencing, the 3-AT treatment had little effect on the I34 modification levels of most of the ADAT-related tRNAs, and only had a minor effect on tRNA^Ala^(AGC) and tRNA^Val^(AAC), indicating that the decrease of tRNA I34 modification in Si*adat2* is due to *adat2* silencing rather than amino acid starvation.

### *adat2* silencing reprograms codon usage-dependent translation elongation kinetics

**Fig 3:**
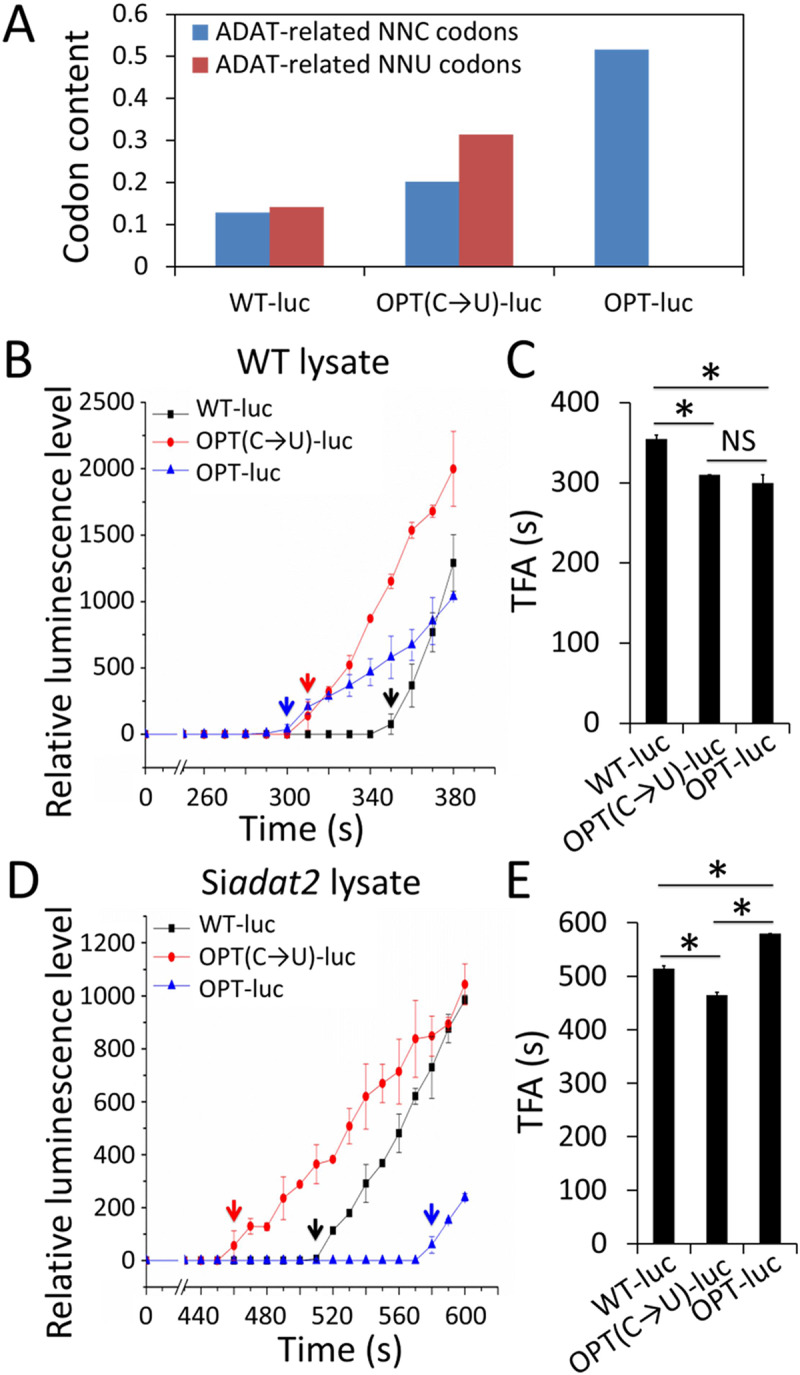
Translation assays *in vitro* demonstrate that *adat2* silencing alters codon optimality for translation elongation. **(A)** Comparison of ADAT-related codon contents (NNC/NNU codon number divided by total codon number) of WT, OPT(C→T), and OPT *Luc* mRNAs. **(B)** Real-time measurement of luciferase activity from WT, OPT(C→T), and OPT *Luc* mRNAs in WT lysate. Reactions were performed at room temperature. Recorded relative light units (RLUs) were plotted versus translation reaction time in 10-s intervals. TFAs of the luminescence signal are indicated by arrows. **(C)** Statistical analysis of TFAs of the signals from the three *Luc* mRNAs in the WT cell lysate. **(D)** Real-time measurement of luciferase activity from the three *Luc* mRNAs in Si*adat2* lysate in the experiments conducted as described in panel B. **(E)** Statistical analysis of TFAs of the signals from the three reporters in the Si*adat2* cell lysate. Data are means ± SD. *, P < 0.05. NS, not significant, as determined by Student's two-tailed t-test. Compared with WT *Luc*, there are 360 codons were optimized in the OPT *Luc*; Compared with OPT *Luc*, there are 173 ADAT-related NNC codons were changed in OPT(C→T) *Luc* (S11 Fig).

The eight ADAT-related NNC codons are the most preferred codons in *Neurospora* and are also the codons with the fastest translation elongation rates within each codon family [31]. To determine how the changes in the ADAT-related tRNA profiles caused by *adat2* silencing would affect mRNA translation elongation kinetics, we used a *Neurospora* cell-free translation system that was previously shown to accurately reflect protein translation kinetics *in vivo* and can determine the effect of codon usage on mRNA translation elongation rate [31,52–56]. Using the same amounts of different versions of firefly *luciferase* (*Luc*) mRNAs with different codon usage profiles as substrates, the *in vitro* translation assays were previously used to compare *Luc* mRNA translation elongation rates by determining the time of first appearance (TFA) of luminescence signal [31]. In this assay, the luminescence signal can be immediately detected when the first round of translation of *Luc* mRNA is finished, so the difference in TFAs reflects the difference in translation elongation rates of the different *Luc* mRNAs. We synthesized the WT-*Luc* mRNA, OPT-*Luc* mRNA (*Luc* ORF codons were optimized to the most preferred codons according to the *N*. *crassa* codon usage table) and OPT(C→T)-*Luc* mRNA (the NNC codons in the eight ADAT-related codon families in OPT-*Luc* mRNA were partially changed to the less preferred synonymous NNT codons) for *in vitro* translation in lysates made from either the WT strain or the Si*adat2* strain (Fig 3A). As expected, in the WT lysate, the TFAs of the OPT-*Luc* and OPT(C→T)-*Luc* mRNAs were both significantly faster than that of the WT-*Luc* mRNA (Fig 3B and 3C). In the Si*adat2* lysate, however, the OPT(C→T)-*Luc* mRNA had the fastest TFA, and the TFA of the OPT-*Luc* mRNA was significantly slower than that of the WT-*Luc* mRNA (Fig 3D and 3E). These results demonstrated that *adat2* silencing resulted in altered codon optimality in translation elongation: compared to the NNC codons, the NNU codons became better in the ADAT-related codon families in the Si*adat2* extracts. Such changes are consistent with the ADAT-related tRNA profile changes observed in the Si*adat2* strain, indicating that the optimal codon selection in translation elongation is determined by the relative abundances of corresponding tRNAs. It should also be noted that the TFAs of all three versions of the *Luc* mRNAs were longer in the Si*adat2* lysate than that in the WT lysate, indicating that *adat2* silencing impaired the translation elongation process.

**Fig 4:**
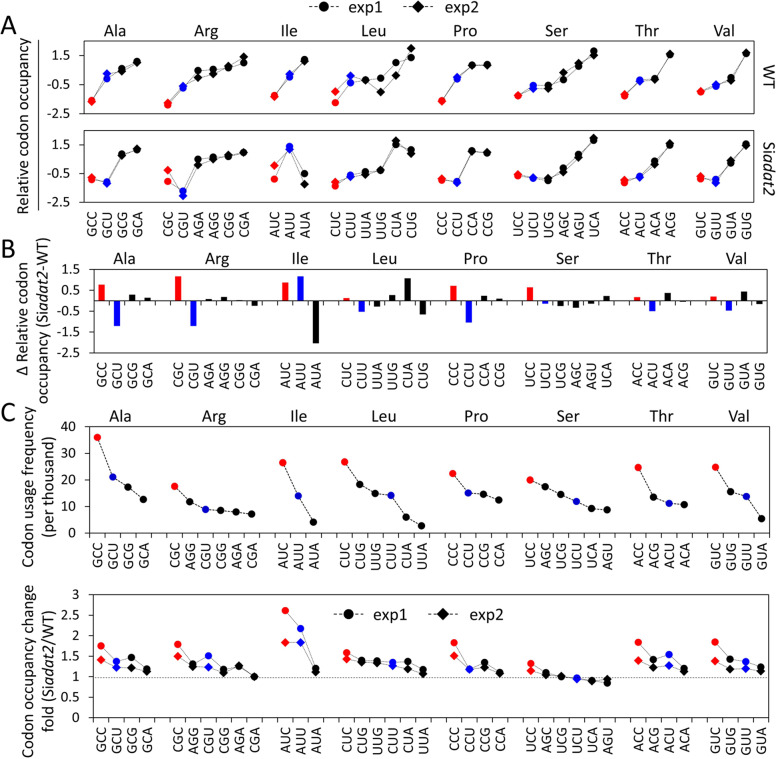
Ribosome profiling demonstrated that *adat2* silencing alters codon optimality in each codon family *in vivo*. **(A)** The relative codon occupancies in each ADAT-related codon family in the WT and Si*adat2* cells. Red and blue colors indicate ADAT-related NNC and NNT codons, respectively. The relative codon occupancy values in each codon family were normalized and centralized by z-score transformation. The results for two independent biological replicates are shown. **(B)** Changes of the relative codon occupancy (Δ relative codon occupancy) in each ADAT-related codon family in the Si*adat2* relative to the WT cells. The ADAT-related eight NNC and NNT codons are marked as red and blue bars, respectively. Data for other codons are in black. Data are averages of two independent biological replicates. **(C)** Genome-wide codon usage frequency (numbers per thousand codons, upper panel) in *N*. *crassa* and codon occupancy change folds (lower panel) in ADAT-related codon families between the Si*adat2* and WT cells. Data from two independent biological replicates are shown. The codon occupancy values are normalized to that of the most occupied codon (5’-CGA-3’, arginine).

Ribosome profiling is a powerful approach for studying mRNA translation dynamics *in vivo* as it provides codon-level resolution of ribosome locations on mRNAs [57,58]. Using ribosome profiling experiments, we previously demonstrated that codon usage plays an important role in regulating translation elongation rate in *Neurospora*, because the codon occupancy levels inferred by the number of ribosome protected fragments (RPFs) on codons inversely correlated with translation elongation velocity [31,34]. To determine the impact of impaired tRNA A34 deamination on the relative decoding rates of individual codons in each family, we performed ribosome profiling experiments in the WT and Si*adat2* strains (S2A and S2B Fig). As shown in S2C Fig, the RPFs mapped to mRNAs exhibited a strong triplet periodicity, indicating that our ribosome profiling experiments were very accurate in identifying the A sites in ribosomes. The results from two independent biological replicates were very consistent and showed very high correlations between them, the Pearson’s correlation coefficients of the relative and absolute codon occupancy values between the two independent biological replicates for both the WT strain and the Si*adat2* strain were all more than 0.96 (Fig 4A and 4C and S3A–S3F Fig). As expected, the relative codon occupancies of the NNC codons within the A site were invariably the lowest within each of the eight ADAT-related codon families in the WT strain (Fig 4A), indicating that they are the optimal codons for translation elongation. In the Si*adat2* strain, however, the codons with lowest relative codon occupancy became the NNT codons within the Ala, Arg, Pro, and Val codon families, suggesting that the NNT but not NNC codons became optimal codons for translation elongation. For the Ser codons, both TCT and TCG codons had lower relative codon occupancy values than the TCC codon.

We then used the difference of the relative codon occupancy (Δ relative codon occupancy (Si*adat2*-WT)) to reflect the relative codon occupancy changes in each codon family in the WT and Si*adat2* strains. With the exception of the Ile(ATT) codon, the relative codon occupancies at the A site were preferentially increased for the NNC codons but were decreased for the NNT codons for seven of the eight ADAT-related codon families (Fig 4B). On the other hand, very few differences in the relative codon occupancies were observed between ADAT-related NNC codons and NNT codons within A+1, P, or E sites in the two strains (S4 Fig), indicating the specificity of our analysis for the A site. In addition, comparison of ribosome profiling results for cultures with/without cycloheximide (CHX) treatment showed that CHX treatment and different glucose concentration conditions had little effect on the relative codon occupancy of ADAT-related NNC and NNT codon families in *N*. *crassa* (S5 Fig), indicating that the changes of the relative codon occupancy of ADAT-related NNT codons are not caused by artificial effects in ribosome profiling experiments.

It should be noted that in addition to specific response, *adat2* silencing also causes secondary effects such as amino acid starvation response (see below) and a general translation repression, which may indirectly affect translation kinetics of the Ile codons and other codons. To exclude the possibility that the specific and predicted ribosome occupancy effects we observed for the ADAT-related codons were caused by global translation inhibition or general amino acid starvation, we performed ribosome profiling under conditions that can also result in amino acid starvation-like response. As shown in S6 Fig, ribosome profiling of the WT strain in lower carbon source medium (lowC), low carbon and no nitrogen medium (lowC & noN) and of the Δlys4 mutant (a strain deficient in lysine biosynthesis cultured in medium with limited lysine) showed that these conditions did not cause significant ribosome occupancy changes for the ADAT-related NNC and NNT codons as we observed when *adat2* was silenced. These results demonstrate that amino acid starvation-like response does not cause the observed ribosome occupancy changes for the ADAT-related NNC and NNT codons.

To compare the decoding rate changes of all codons in the WT strain and Si*adat2*, the absolute ribosome codon occupancy between the Si*adat2* and the WT strains was determined (see Methods for details). The fold-change of absolute ribosome codon occupancy indicated that the most preferred NNC codons in almost all ADAT-related codon families showed largest fold increases upon *adat2* silencing (Fig 4C). Together these and the in vitro translation kinetics results indicate that *adat2* silencing had specific but differential effects on ribosome decoding rates for ADAT-related codons: slows down translation speed for NNC codons but increases the relative translation speed for seven out of eight NNT codons, resulting in changes of codon optimality for ribosome decoding rates of ADAT-related NNC codons.

Our result also showed that *adat2* silencing also affected translation elongation kinetics of non-ADAT-related codon families to a less extent (S7 Fig). This is most likely due to an indirect secondary effect of amino acid starvation response induced by *adat2* silencing (see below), which was known to influence the charging levels of different tRNA species [59]. Together, these results demonstrated that *adat2* silencing reprograms the translation elongation kinetics of the ADAT-related codons.

### *adat2* silencing causes genome-wide codon usage-biased ribosome pausing on mRNAs

**Fig 5:**
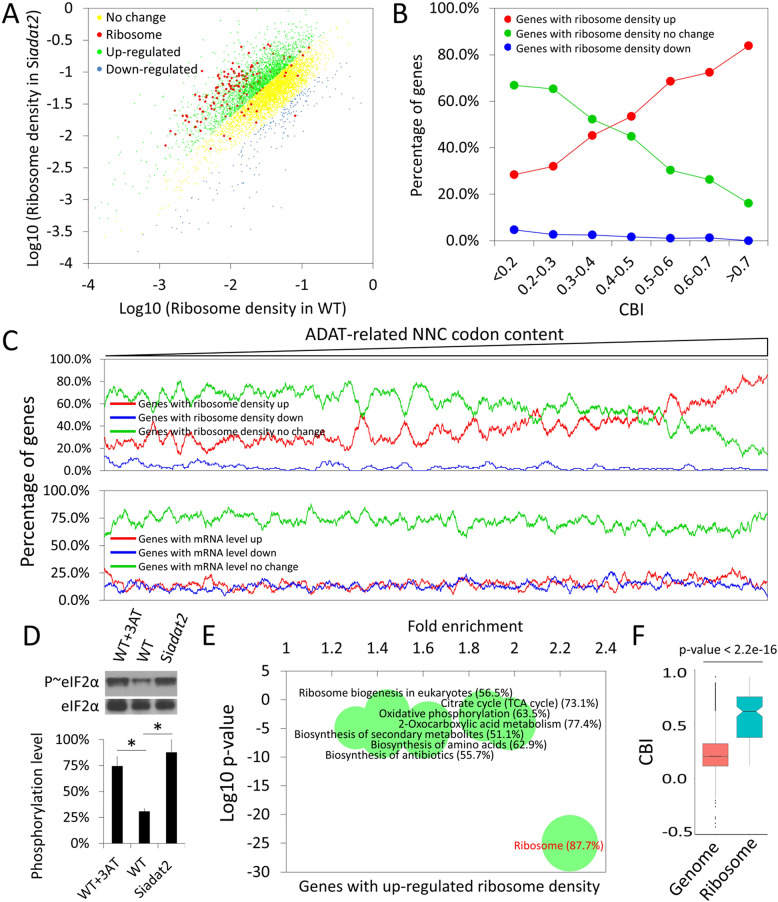
Codon usage-biased ribosome pausing after *adat2* silencing. **(A)** A scattered plot showing the ribosome density of each gene in the WT strain versus the Si*adat2*. The genes with up-regulated, down-regulated, and unchanged ribosome density in the Si*adat2* compared to the WT strain are indicated by green, blue, and yellow dots, respectively. RPGs are marked as red dots. **(B)** Line plot showing CBI values versus percentages of genes with up-regulated, down-regulated, and unchanged ribosome densities in the Si*adat2* compared to the WT strain. **(C)** Upper panel: Percentage of genes with up-regulated ribosome density in the Si*adat2* strain increased with the increase of the ADAT-related NNC codon contents. Lower panel: No correlation was observed between the mRNA level change and the ADAT-related NNC codon contents. The mRNA level of each gene was measured by their average RPKM values of two independent mRNA-seq experiments. All the detected genes were ranked by their ADAT-related NNC codon contents, a window containing 100 genes slides from low to high ADAT-related NNC codon contents. The red, blue and green curves represent the percentages of genes with up-regulated, down-regulated and non-changed ribosome density (upper panel) or mRNA level (lower panel) in each window, respectively. **(D)** Representative western blot for eIF2α and phosphorylated eIF2α in the lysates of the WT cells treated with 3-AT, the WT cells without any treatment, and the Si*adat2* cells in the presence of QA. The phosphorylation levels were represented by the ratios of phosphorylated eIF2α normalized to eIF2α. The graph shows quantification of means ± SD (n = 3). *, P < 0.05, as determined by Student's two-tailed t-test. **(E)** Functional enrichment analysis of genes with up-regulated ribosome density in the Si*adat2* compared to the WT strain. Genes with up-regulated ribosome density were used as the probe and the whole genome was used as background. Bubble sizes represent the percentages (% in parentheses) of enriched genes among total genes in each pathway. Representative functional categories from KEGG database are presented; for a complete list, see S1 Table. **(F)** Boxplot of CBI values of the whole genome and that of the RPGs. The p-value is determined by Welch’s two-tailed t-test.

To further determine the impact of the ADAT-related tRNA profile changes on ribosome traffic on mRNAs, we calculated ribosome density (the number of RPFs normalized by library size, gene length and mRNA level, see Methods for details) at the individual gene level. We found that, compared to the WT strain, the majority of mRNAs in the Si*adat2* strain showed increased ribosome density. Ribosome density was increased on approximately 48.8% (an average for two independent biological replicates) of genes (fold change for Si*adat2*/WT > 2), and only a small proportion (4.9%, an average for two independent biological replicates) of genes had decreased ribosome density (fold change for Si*adat2*/WT < 0.5) (Fig 5A and S8 Fig).

Because CBI values of genes tightly correlate with ADAT-related NNC codon contents, we determined the genome-wide correlation between the ribosome density of genes and their CBI values. When genes with detectable ribosome density were divided into seven groups by their CBI values, we found that the proportion of genes with up-regulated ribosome density increased progressively as CBI values increase, while the opposite occurred for genes with down-regulated ribosome density (Fig 5B). Such a correlation is not caused by uneven distribution of gene numbers in each group or a bias of CBI calculation for non-ADAT-related codons, since the same correlation could be observed in continuous scanning windows containing the same number of genes with gradually increasing ADAT-related NNC codon contents (Fig 5C, upper panel). Additionally, we used mRNA-seq results (see below) to analyze the relationship between the ADAT-related codon usage and mRNA level changes in the WT and Si*adat2* strains. We found that the ADAT-related NNC codon contents do not correlate with the mRNA level changes (Fig 5C, lower panel), indicating that codon usage/translation kinetics does not play a major role in determining mRNA level changes in response to *adat2* silencing.

The increase of ribosome occupancy for ADAT-related NNC codons suggests that ribosomes may pause or stall on these codons in Si*adat2* (Fig 4C). Previous ribosome profiling studies demonstrated that the ribosome density of a gene correlates positively with translation initiation and negatively with translation elongation [31,60,61]. Since *adat2* silencing resulted in severe growth defect and impaired *in vitro* translation efficiency (Fig 3B–3E), the observed increase of ribosome density in the Si*adat2* strain should not be caused by a global increase of translation efficiency. In *Neurospora*, amino acid starvation can be induced by the addition of 3-AT [50,51]. Growth of the WT strain with 3-AT and *adat2* silencing both resulted in a significant induction of eIF2α phosphorylation (Fig 5D). Phosphorylation of eIF2α is known to be induced upon amino acid starvation; the phosphorylation of this factor mediates translation initiation repression [62,63]. This result suggests that both amino acid starvation and *adat2* silencing result in a similar stress response. Therefore, the ribosome density increase in the Si*adat2* strain should not be due to increased translation initiation rate as indicated by an increase of eIF2α phosphorylation level in the Si*adat2* strain. Furthermore, with the increase of ADAT-related codon contents, the percentage of genes with up-regulated ribosome density increased (Fig 5C), while the percentage of genes with down-regulated translation efficiency increased (see below). These results suggest that *adat2* silencing resulted in an imbalance between the ADAT-related tRNA profile and gene codon usage to cause genome-wide codon usage-biased ribosome pausing on mRNAs, that is, ribosomes preferentially paused on the mRNAs with more preferred codons (such as the NNC codons) after *adat2* silencing.

Gene functional enrichment analysis based on the ribosome density changes indicated that genes encoding the protein components of ribosomes were the most dramatically enriched among genes with up-regulated ribosome density in the Si*adat2* strain (Fig 5E and Supplementary S1 Table). The ribosomal protein genes (RPGs) are also one of the functional categories with much higher average CBI values than that of the genome average (Fig 5F), consistent with the codon usage-biased effect of ribosome pausing on translation elongation. In contrast to their increased ribosome density, the protein levels of most RPGs were decreased upon *adat2* silencing (see below). These results suggest that mRNA translation elongation rates of RPGs were strongly repressed by *adat2* silencing.

### *adat2* silencing changes proteome landscape in a codon usage-biased manner

**Fig 6:**
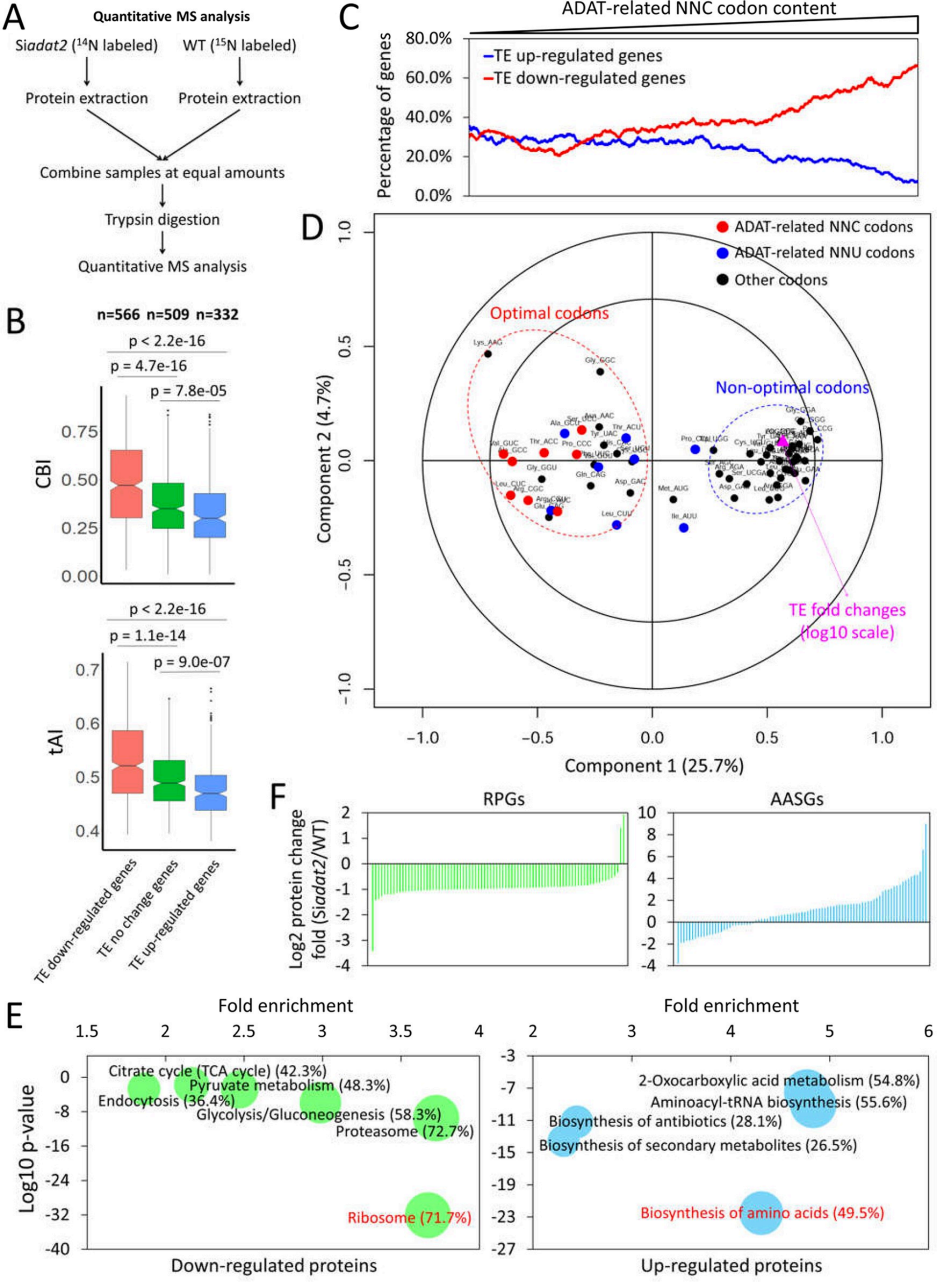
*adat2* silencing resulted in codon usage-biased proteome changes. **(A)** A diagram depicting the experimental procedures for the quantitative MS analysis of protein level changes in the Si*adat2* compared with the WT strain. **(B)** Boxplots of CBI (upper panel) and tAI values (lower panel) for genes whose TEs were up-regulated, down-regulated, and unchanged in the Si*adat2* compared with the WT strain. n = gene numbers in corresponding groups. The p-value is determined by Welch’s two-tailed t-test. **(C)** Line plot showing the percentage of genes with up/down-regulated TEs in the Si*adat2* strain as the ADAT-related NNC codon contents increase. These genes were ranked by their ADAT-related NNC codon contents and the percentages of genes were plotted in a sliding window (200 genes/window) from low to high ADAT-related NNC codon contents. **(D)** PLSR analysis of the effect of codon content on protein level changes. Genes with changed TE levels were used. The log10 (TE fold change) was used as the response (Y) and the 61 codon contents were used as the regressor (X). The model has 2 components, and includes leave-one-out (LOO) cross-validated predictions [93]. The outer and inner circles indicate 100% and 50% explained variance, respectively; 30.4% of observed variance can be explained by two latent components (ncomp = 3, component 1: 25.7%, component 2: 4.7%). The purple triangle represents the TE fold change. **(E)** Functional enrichment analysis of down-regulated (left) and up-regulated (right) proteins in the Si*adat2* compared with the WT strain. Genes with up- and down-regulated protein levels were used as probes, respectively, and the whole genome was used as background. Bubble sizes represent the percentages (% in parentheses) of enriched genes among total genes in each pathway. Representative functional categories from KEGG database are presented; see S1 Table for a complete list. **(F)** Change fold of the protein levels of individual ribosomal proteins (left) and proteins involved in amino acid biosynthesis (right) in the Si*adat2* strain compared with the WT strain.

To determine the impact of codon usage-biased ribosome pausing on protein landscape, we performed genome-wide proteome quantification by quantitative mass spectrometry (MS) analyses [64]. The WT strain was cultured in medium with ^15^NH_4_Cl as the sole nitrogen source and the Si*adat2* strain was cultured in the non-isotope-containing medium (^14^NH_4_Cl), so that the proteins from the WT strain and Si*adat2* were labeled with ^15^N and ^14^N, respectively. The protein extracts of these two strains were mixed at a ratio of 1:1 before trypsin digestion and subjected to MS analyses (Fig 6A). The ^14^N/^15^N ratios of identified proteins, which reflected the relative protein level changes, were determined. The Pearson’s correlation coefficient of the results from the two independent biological replicates was 0.83 (S9A Fig and S2 Table). The ^14^N/^15^N ratios of 1878 *Neurospora* proteins could be detected in two independent experiments. Of these proteins, levels of 1658 (88.3%) proteins were changed in the same trend in both of the duplicate samples (S2 Table), and the averages of the ^14^N/^15^N ratios of these proteins were used for further analyses. To identify proteins with marked level changes from the control, we set 1.5 (Si*adat2*/WT > 1.5) and 0.67 (WT/Si*adat2* > 1.5) of the ^14^N/^15^N ratios as the cutoff values for proteins that were either up- or down-regulated due to *adat2* silencing, respectively. According to this criterion, 395 and 594 proteins were up-regulated and down-regulated, respectively, in the Si*adat2* strain (S2 Table).

To determine the effect of codon usage on protein level changes at the translational level independent of mRNA level change, we defined the protein level fold change (^14^N/^15^N ratios) divided by mRNA level fold change between the Si*adat2* and WT strains as the translation efficiency (TE) fold change. Similar to protein fold changes, we set 1.5 and 0.67 of the TE fold change as the cutoff values to identify genes with TEs that were either up- or down-regulated due to *adat2* silencing, respectively. As shown in Fig 6B, the genes with up-regulated TEs were generally those with more non-optimal codons, as indicated by their significantly lower CBIs or tRNA adaptation indexes (tAIs) [65]. On the other hand, the genes with down-regulated TEs tended to have significantly higher CBI and tAI values. Opposite to the ribosome density results (Fig 5C, top panel), as the ADAT-related NNC codon contents increased, the proportion of genes with down-regulated TEs increased, while the proportion of genes with up-regulated TEs decreased (Fig 6C). These results suggest that *adat2* silencing affected protein level change at translational level in a codon usage-dependent manner: it suppressed protein expression of genes with more preferred codons and promoted those with more non-preferred codons.

To determine the contributions of all individual codons to TE changes after *adat2* silencing, partial least squares regression (PLSR) analysis was performed to explore the relationship between codon content and TE fold changes on all TE changed genes. As shown by the correlation-loading plot in Fig 6D, we found that the optimal codons and non-optimal codons clustered together, respectively, and can be separated by their positions in the first component direction, indicating that the first component largely reflects the relative codon optimality in each codon family. Importantly, the non-optimal codons clustered together with the TE fold change, while the optimal codons were segregated from the TE fold change in the first component direction (Fig 6D), indicating that the optimal codon content anti-correlates with the TE fold change, while the non-optimal codon content correlates with the TE fold change. This result further suggests that the TEs of genes containing more optimal codons (such as NNC codons) were more likely to be down-regulated upon *adat2* silencing than those with less optimal codons. This conclusion is consistent with the ribosome profiling results, which indicated that *adat2* silencing preferentially paused at optimal codons. Therefore, the codon usage-biased pausing on mRNAs differentially affects translation efficiency of mRNAs, resulting in codon usage-dependent protein landscape changes. In our PLSR analysis, the Lys AAG codon is the codon exhibiting the strongest effect on the protein level changes (Fig 6D). This result is likely due to that AAG is the most abundant codon in *Neurospora* genes detected by our quantitative MS, and it often co-occurs with the ADAT-related NNC codons in genes. As a result, the PLSR analysis cannot distinguish its contribution from that of the ADAT-related NNC codons.

Gene functional enrichment analysis based on protein level differences in the Si*adat2* and WT strains revealed that RPGs were the most significantly enriched among the down-regulated proteins, whereas those involved in amino acid biosynthesis (AASGs) were the most significantly enriched among the up-regulated proteins in the Si*adat2* strain (Fig 6E and Supplementary S1 Table). Our quantitative MS data showed that, compared with the WT strain, the protein levels of 73/75 ribosomal proteins were decreased in the Si*adat2* (Fig 6F, left panel). These results are consistent with the ribosome profiling results showing that this group of genes are the most significantly enriched among those with increased ribosome density (Fig 5E and Supplementary S1 Table), suggesting that their reduced protein levels in the Si*adat2* are due to increased ribosome pausing on their mRNAs. On the other hand, the protein levels of most of genes in the group of AASGs increased in the Si*adat2* (Fig 6F, right panel). Although the codon biases of AASGs is not as high as that of RPGs, the codons of AASGs are more optimized than the genome average, suggesting that there is another mechanism regulating their expression.

### CPC-1 mediates a transcriptional response upon *adat2* silencing to counteract the global repression of translation

The up-regulation of AASGs upon *adat2* silencing is reminiscent of an amino acid starvation response, in which, a cross-pathway control protein 1 (CPC-1), the *Neurospora* ortholog of the yeast bZIP transcription factor GCN4, is a critical transcription activator of AASGs [66–68]. Amino acid starvation leads to activation of many genes in multiple amino acid biosynthetic pathways, called cross-pathway way control in *Neurospora* [62,69,70]. Similar to GCN4 in yeast, CPC-1 binds to the 5’- TGACTCA-3’ motifs in target gene promoters to activate transcription [62,67,68,71]. The upstream open reading frames (uORFs) in the yeast *GCN4* regulate protein expression at the translational level [62,71]. Under normal conditions, the translation of the uORFs prevents translation initiation from the downstream *GCN4* ORF, resulting in the suppression of GCN4 expression. Under amino acid starvation condition, however, the scanning 40S ribosomes bypass the uORFs and initiate translation at the downstream *GCN4* ORF, resulting in the induction of GCN4 protein expression. CPC-1 level is normally very low and not always detected by MS in the WT strain. However, in one of the two independent experiments, CPC-1 was detected in both strains and found to be up-regulated ~130 fold in the Si*adat2* strain. The dramatic increase in CPC-1 level could not be explained by ~3 fold increase of *cpc-1* mRNA level in the Si*adat2* strain revealed by the mRNA-seq result (S3 Table), suggesting the involvement of posttranscriptional regulation for CPC-1 expression. Like *GCN4*, the *cpc-1* gene also has uORFs that are likely involved in regulating *cpc-1* mRNA translation upon amino acid starvation [72]. The strong induction of CPC-1 expression by *adat2* silencing suggests that the ADAT-related tRNA profile changes triggered an amino acid starvation condition and CPC-1 is a major factor that mediates the transcriptional response of its target genes.

**Fig 7:**
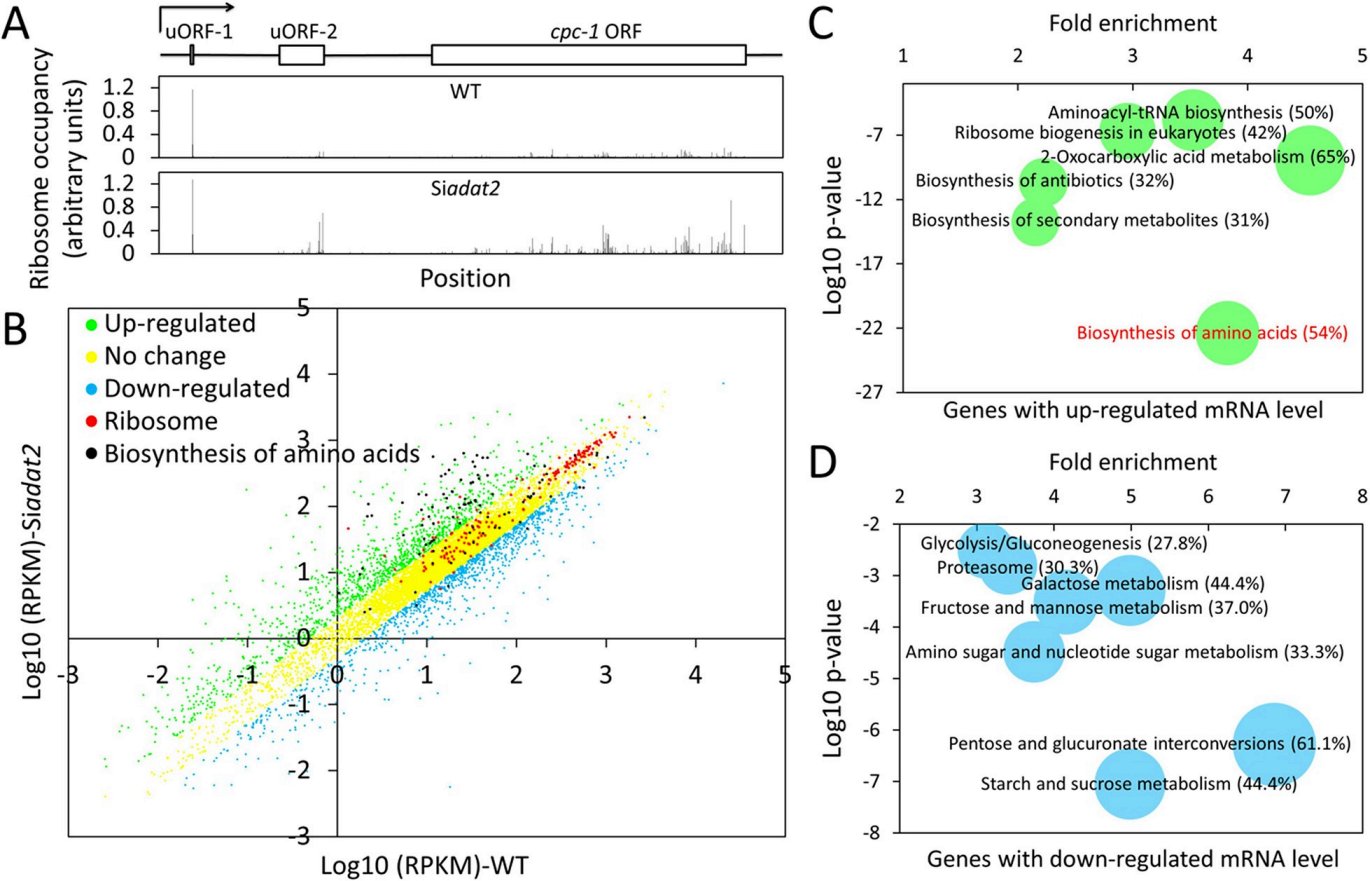
Transcriptional responses upon *adat2* silencing. **(A)** The ribosome occupancy of *cpc-1* in the WT and Si*adat2* strains across the entire transcript. A schematic of the transcript is shown above the plots. The numbers of RPFs on each codon of *cpc-1* transcript were normalized by the RPF library size and its mRNA level. **(B)** Comparison of the transcript expression profiles of the Si*adat2* and WT strains. **(C** and **D)** Gene functional enrichment analysis based on the mRNA level changes for the up-regulated genes (C) and down-regulated genes (D). Genes with up- and down-regulated mRNA levels were used as probes respectively, and the whole genome was used as background. Bubble sizes represent the percentages (% in parentheses) of enriched genes among total genes in each pathway. Representative functional categories from KEGG database are presented; for a complete list, see S1 Table.

To examine the posttranscriptional regulation of *cpc-1*, we compared the ribosome profiling results of the *cpc-1* mRNA between the WT and Si*adat2* strains. The strong induction of CPC-1 expression was indeed confirmed by the ribosome profiling result. In the WT strain, most of the ribosomes were found on the uORF-1 of the *cpc-1* mRNA (a 2-codon region), and very few ribosomes were associated with its uORF-2 (41 codons) or ORF regions (Fig 7A). Thus, *cpc-1* translation is suppressed under normal conditions by uORF-1, which prevents translation initiation at the downstream *cpc-1* ORF. In the Si*adat2* strain, however, the ribosome occupancy at uORF-2 and the *cpc-1* ORF were both dramatically induced. The translational regulation of *cpc-1* by uORFs in WT strain was also previously observed by ribosome profiling experiments [73]. These results indicate that, as *GCN4* in yeast [62,71], translational control mediated by uORFs also plays an important role in regulating CPC-1 expression by allowing ribosomes to bypass the uORFs and reinitiate at the downstream *cpc-1* ORF in the Si*adat2* strain.

To determine the mRNA level changes between the Si*adat2* and WT strains, we performed high-throughput mRNA sequencing experiments. The correlation between the two independent biological replicates is very high, with the Pearson’s correlation coefficient 0.99 and 0.98 for the WT strain and the Si*adat2*, respectively (S9B and S9C Fig and S3 Table). Analyses of the mRNA-seq results comparing the transcriptomes of the WT and Si*adat2* strains showed that the mRNA levels of 1219 and 1049 genes were up-regulated (RPKM (Reads Per Kilo base Per Million) fold change for Si*adat2*/WT > 2) and down-regulated (RPKM fold change for Si*adat2*/WT < 0.5) in the Si*adat2* strain, respectively (Fig 7B and S3 Table). Gene functional enrichment analysis based on the gene annotation of KEGG database [74] of the up-regulated genes revealed that genes involved in biosynthesis of amino acids, 2-oxocarboxylic acid metabolism, and aminoacyl-tRNA biosynthesis were among the most significantly enriched groups (Fig 7C and Supplementary S1 Table). On the other hand, many functional categories related to energy metabolism were enriched among the down-regulated genes (Fig 7D and Supplementary S1 Table). The induction of genes associated with AASGs and tRNA biosynthesis is consistent with a transcriptional response due to impaired tRNA processing or amino acid starvation [68,75]. Of the detected 97 AASGs, the mRNA levels of 56 genes were up-regulated more than 2-fold in the Si*adat2* strain (Fig 7B), indicating that, in addition to the regulation of protein expression at translational level, *adat2* silencing also triggers a transcriptional response that promotes amino acid synthesis. It is also important to note that the mRNA levels of most of RPGs were not affected by *adat2* silencing (Fig 7B), suggesting that the suppression of ribosomal protein expression we observed in the Si*adat2* is mainly mediated by reduced translational efficiency.

Microarray-based experiments were previously performed to compare the transcriptional profiles of WT and *cpc-1* mutant exposed to 3-AT in *Neurospora* [68]. We found that of the 216 genes that were induced more than 2-fold by 3-AT in the WT strain in the microarray experiments, 115 (53%) were also induced more than 2 fold upon *adat2* silencing in our mRNA-seq results. Moreover, among the 187 direct or indirect target genes of CPC-1 previously identified (their induction by 3-AT was either abolished or significantly reduced in the *cpc-1* mutant), the mRNA levels of 82 (43%) genes were also induced by *adat2* silencing. These genes include some known targets of CPC-1 such as *trp-1*, *arg-12*, *his-3*, *trp-3* and *leu-6*. These results indicate that the induction of CPC-1 plays a critical role in mediating the transcription response of its targets upon *adat2* silencing.

## Discussion

Codon usage plays a major role in controlling gene expression levels at both transcriptional and translational levels. At the translational level, the correlation between tRNA abundance and codon usage bias was proposed to regulate translation efficiency [14,27,65,76]. Paradoxically, most eukaryotic organisms lack the GNN tRNA genes but exhibit codon usage biases for C-ended codons. In this study, we showed *in vivo* that the A34-to-I editing of tRNAs by ADAT allows codon usage-biased protein expression in *Neurospora*. Nearly all of the ADAT-related tRNAs with ANN anticodons are modified by A-to-I editing at the 34^th^ position, and the ADAT is required for such modifications. We showed that *adat2* silencing led to dramatic changes in the ADAT-related tRNA profile in cells, which resulted in genome-wide codon usage-biased ribosome pausing on mRNAs and corresponding changes in the protein landscape in cells. These results highlight the importance of tRNA A34-to-I editing in driving codon usage-biased translation and the role of codon usage in determining proteome profile. Thus, our results provide strong genetic evidence in support of the hypothesis explaining the previously observed correlations between tRNA contents and codon usage biases in bacteria and eukaryotes [35].

It was previously suggested that mRNAs with stretches of ADAT-related codons are more dependent on I34-tRNAs for their translation (10–11). In addition, I34 modification efficiency during different cell developmental stages may be dynamic [47], indicating that the translation regulation by tRNA I34 modification during cell growth and differentiation is complex. The roles of tRNA A34I modification in modulating codon usage-dependent ribosome decoding rates and protein translation established here by genetic, biochemical and bioinformatics analyses in this study suggest that tRNA A34I modification play an important role in shaping protein landscape in diverse organisms and at different developmental stages.

The silencing of *adat2* expression triggered a response similar to amino acid starvation: both repressed the expression of RPGs and promoted the expression of AASGs (Fig 6E). We also found that the *Neurospora* ortholog of yeast GCN4, CPC-1, is a key mediator of the transcriptional response of AASGs to *adat2* silencing (Fig 7). Similar to that the amino acid starvation triggers GCN4p induction in yeast [62,71], the *adat2* silencing induced the expression of CPC-1 at the translational level. Interestingly, *adat2* silencing also resulted in an increase of translation initiation events at uORF2 of *cpc-1*. To determine whether a similar observation is also seen for yeast *GCN4*, we analyzed two previous ribosome profiling results, in which *GCN4* translation was activated by the addition of 3-AT or deletion of a gene (*kti11*) involved in tRNA modification [77,78]. As shown in S10 Fig, both 3-AT treatment and *kti11* deletion resulted in a dramatic increase of translation from *GCN4* ORF without a decrease of translation initiation from the uORFs, but with an increased translation from either uORF3 or uORF4. Together, these results suggest that an overall increase of translation initiation events on *GCN4* and *cpc-1* mRNAs plays an important role in activating their translation.

Protein synthesis is one of the most energy consuming processes in cells [79]. The codon usage of both of RPGs and AASGs is more optimized than the average of genomes in almost all organisms. When cells encounter extracellular or intracellular stress conditions, the rebalance of various biological processes may be critical for cell survival. Under stress conditions, cell survival has a priority over cell proliferation, so that the synthesis of RPGs is repressed to decrease global translation activity while the synthesis of AASGs is activated to promote cell survival. During this transition, CPC-1 plays a critical role in adapting cells to stress conditions.

In addition to the I34 modification of tRNAs, the wobble bases of several Leu, Thr, Lys, Gln, and Glu tRNAs have also been shown to be modified, and these modifications may influence the translation of codon-biased transcripts [4,5,80]. Moreover, changes in tRNA expression profiles in different growth conditions and tissues have also been implicated in the regulation of protein expression [41,81–83]. Together, these results suggest that tRNA modifications and the regulation of tRNA expression represent an important mechanism to control gene expression in eukaryotic organisms.

## Materials and methods

### Strains and growth conditions

*N*. *crassa* strain 301–6 (*bd*, *A*, *his-3*) was used as the host strain for *his-3*-targeting *adat2* silencing construct. 87–3 (*bd*, *a*; clock WT) was used as the control in this study. Liquid cultures were grown in 0.1% glucose medium (1 × Vogel’s, 0.1% glucose and 0.17% arginine) unless otherwise specified. Race tube medium contained 1 × Vogel’s, 0.1% glucose, 0.17% arginine, 50 ng ml^-1^ biotin and 1.5% agar. All strains were cultured on slants containing 1 × Vogel’s, 2% sucrose and 1.5% agar before performing various experiments. All the strains were cultured under constant light at room temperature.

### Plasmid constructs and *adat2* silencing

To generate the inducible silencing construct of *adat2*, an 801 bp segment was amplified with the paired primers 5′-CCATCGATCGCAACCACATCTCAATCGCAAT-3′ and 5′-CGGAATTCCTACAATCGAAACCAGTTAGCAAC-3′ and a 733-bp segment was amplified with the paired primers 5′-TCCCCCGGGCGCAACCACATCTCAATCGCAAT-3′ and 5′-CGGAATTCCATATCCAAAGCCTCCCTCATG-3′. These two segments were the same except that the terminus of the former contains a 68-bp intron sequence of *adat2*. This sequence has no similarity with any other regions of the *Neurospora* genome. These two segments were then ligated into a plasmid named qa.5myc.6His in an opposite orientation. The construct was then integrated into the genome at the *his-3* locus through electrotransformation. Transcription of the long dsRNA was under the control of the inducible *qa-2* promoter. The *adat2*-specific dsRNA and siRNA can be induced by the addition of QA (1×10^−2^ M). Homokaryon strains were obtained by microconidia purification.

### Quantitative MS analysis and database search

For quantitative MS analysis, fresh conidia of the WT strain and Si*adat2* were cultured in liquid medium without arginine (1 × Vogel’s, 0.1% glucose) and with 10^−2 ^M QA (pH = 5.8). To label the proteins in the WT strain and Si*adat2*, ^15^NH_4_Cl and ^14^NH_4_Cl (Cambridge Isotope Laboratories) were used to replace NH_4_NO_3_ in the Vogel’s medium, respectively. The fresh conidia were cultured in plates for three days and then the cultures were cut into small discs. The discs were cultured in the identical medium with orbital shaking (200 rpm) for one more day before sample collection. Total proteins were extracted and precipitated with ten volumes of pre-chilled (-20°C) acetone overnight. The samples were then centrifuged at 14000g for 20 minutes at 4°C before washing the pellets with pre-chilled acetone three times. Approximate 100 μg acetone-precipitated protein pellets were suspended with 200 μl of 200 mM triethyl ammonium bicarbonate, and the ^14^N and ^15^N protein samples were combined at equal protein concentration. To digest the proteins, 4 μg of trypsin per 100 μg of proteins was added, and the samples were incubated overnight at 37°C. The resulting tryptic peptides were desalted and fractionated into eight fractions with Thermo Scientific Pierce High pH Reversed-Phase Peptide Fractionation Kit.

Each fraction was separated over analytical capillary columns (50 μm × 15 cm) packed with 5-μm spherical C18 reversed phase material (YMC). A Waters nanoAcquity UPLC system was used to generate the following HPLC gradient: 0–30% B in 75 min, 30–70% B in 15 min, 70–90% B in 5 min (A: 0.1% formic acid in water, B: 0.1% formic acid in acetonitrile). The eluted peptides were sprayed into Q Exactive mass spectrometer (Thermo Scientific) equipped with a nano-ESI ion source. The mass spectrometer was operated in data-dependent mode with one MS scan followed by ten high-energy collisional dissociation scans for each cycle. Database searches were performed on an in-house Mascot server (Matrix Science Ltd.) against the *Neurospora* protein database. The search parameters were set as follows: 7 ppm mass tolerance for precursor ions; 0.02 Da mass tolerance for product ions; three missed cleavage sites were allowed for trypsin digestion; enzyme was chosen as semi-trypsin which allows non-specific cleavage at peptide N-terminus; protein N-terminal acetylation, methionine oxidation, cysteine carbamidomethylation variable modifications were included; quantification method was defined as ^15^N Metabolic. The search results were filtered with both peptide significance threshold and expectation cutoff value of 0.05. The quantification of the ratios of ^14^N/^15^N peptide pairs was performed automatically by Mascot Distiller. The average is the geometric mean and the standard deviation is geometric standard deviation SD (geo). Only data with SD (geo) < 1000 was retained for further analysis.

### Quantitative reverse transcription PCR (qRT-PCR)

For qRT-PCR, the culture conditions are as follows: fresh conidia (one week post inoculation on slants) of the WT strain and Si*adat2* were cultured in 50 ml liquid growth medium (1 × Vogel’s, 0.1% glucose and 0.17% arginine) with 10^−2^ M QA (pH = 5.8) in plates at room temperate for three days. The cultures were cut into small discs with a diameter of 1 cm, and then the discs were transferred into flasks with the same liquid medium and were grown with orbital shaking (200 rpm) for one more day before sample collection. RNA extraction and qRT-PCR were performed as previously described [84]. For qRT-PCR, the *N*. *crassa* gene coding for β-tubulin was used as an internal control. The primer pairs 5′-CCGAACTTGAGACGCCTGAGGA-3′, 5′-CCAGAGTAGGACAGCAGAGCACAA-3′and 5′-GCGTATCGGCGAGCAGTT-3′, 5′-CCTCACCAGTGTACCAATGCA-3′ were used to amplify the *adat2* and β-tubulin gene, respectively.

### Phosphorylation level assay of eIF2α

To examine the phosphorylation level of eIF2α, fresh conidia of the WT strain and Si*adat2* were cultured in 50 ml liquid growth medium (1 × Vogel’s, 0.1% glucose and 0.17% arginine) with 0.5×10^−2 ^M QA (pH = 5.8) in plates at room temperate for two days. The cultures were cut into small discs and were cultured in the same liquid medium with orbital shaking (200 rpm) for 10 h before sample collection. 3-aminotriazole (3-AT, a final concentration of 5mM) was added in the 3-AT treated WT sample. Protein extraction and western blot analysis were performed as previously described [85]. Phosphatase inhibitors (25mM NaF, 10mM Na_4_P_2_O_7_.10H_2_O, 2mM Na_3_VO_4_, and 1mM EDTA) were added to protein extraction buffer. PVDF membrane was blocked with PBST containing only 5% bovine serum albumin. The primary antibodies used for detecting the phosphorylation level of eIF2α and protein level of eIF2α were recombinant Anti-EIF2S1 (phospho S51) antibody [E90] (abcam, Cat NO.: ab32157) and Anti-EIF2S1 antibody (abcam, Cat NO.: ab137626), respectively. Densitometry was performed using Image J.

### Codon manipulation, indices calculation and data collection from databases

The codons of luciferase gene were optimized or de-optimized based on the *N*. *crassa* codon usage frequency from the Codon Usage Database (https://www.kazusa.or.jp/codon/cgi-bin/showcodon.cgi?species = 5141). The mutated sites for the optimized or de-optimized versions of luciferase gene are shown in S11 Fig. The CBI and tAI were calculated using CodonW [19] and stAIcalc [86], respectively. The tRNA copy number-related data of 959 organisms was collected from GtRNAdb database (http://gtrnadb.ucsc.edu/GtRNAdb2/). The codon usage frequency data of the 959 organisms was collected from Codon Usage Database (https://www.kazusa.or.jp/codon/). The ADAT-related NNC codon content for each gene was determined by the total number of the eight ADAT-related NNC codons divided by the total codon number in this gene.

### Preparation of *N*. *crassa* cell free lysates, *in vitro* translation, and *in vitro* luciferase reporter assay

For preparation of *N*. *crassa* cell free lysates, fresh conidia of the WT strain and Si*adat2* were cultured in liquid medium (1 × Vogel’s, 0.1% glucose, 0.17% arginine, and 10^−2 ^M QA (pH = 5.8)) in plates for two days and then the cultures were cut into small discs. The discs were cultured in liquid medium (1 × Vogel’s, 0.1% glucose, 0.17% arginine and 5×10^−3 ^M QA (pH = 5.8)) with orbital shaking (200 rpm) for 16 h before sample collection. The *N*. *crassa* cell free lysates were prepared as previously described [31,56]. To prepare the mRNA templates for *in vitro* translation, the *in vitro* transcription method was used as previously described [31]. The *in vitro* translation approach and luciferase reporter assay were the same as previously described [31] except that the luminescence signals were recorded continuously in 10-s intervals.

### Ribosome profiling

For ribosome profiling, the culture conditions of the WT and Si*adat2* strains are the same as described in the section of qRT-PCR experiment. CHX (final concentration of 50 μg/ml) was added to stabilize ribosomes prior to footprinting 10 min before sample collection. To rule out the possible effect of CHX treatment or different concentrations of glucose in medium, the ribosome profiling results of the WT strain cultured in 0.1% and 2% glucose medium without CHX treatment were also used to compare with that treated with CHX. All samples were separated into two parts: one part was used for ribosome profiling and the other part was used for accompanying RNA-seq, which was used to normalize the RPFs data. The ribosome profiling was performed using the protocol for ARTseq Ribosome Profiling Kit (Yeast) as described previously [31]. CHX (final concentration of 0.1 mg/ml) was also added at the RNA extraction step of all samples. We did not perform the Ribo-Zero depletion of rRNA; the reads from rRNA contamination were deleted by the downstream bioinformatics analysis. All the libraries were subjected to Illumina HiSeq2000 sequencing. The result indicated CHX treatment and different concentrations of glucose did not have a significant effect on the relative codon occupancy in *Neurospora* (S5 Fig), which is consistent with the results from previous research [87,88].

### Bioinformatics analysis of ribosome profiling results

For both of the ribosome profiling and the accompanying RNA-seq experiment, the adaptors in raw reads were removed and the reads shorter than 25 nt were discarded. In the ribosome footprint libraries, for the first experiment, the total reads were 89.58 million and 92.48 million for the WT strain and Si*adat2*, respectively. For the second independent biological replicate, the total reads were 76.50 million and 138.43 million for the WT strain and Si*adat2*, respectively. After removing the rRNA sequences and tRNA sequences, the remaining reads were mapped to the *N*. *crassa* reference sequences (coding sequences with upstream 100 bp and downstream 100 bp) using Bowtie2. One mismatch in the seed sequence was allowed during mapping. Only reads with unique match to the reference were retained for further analysis. The majority of footprint reads aligning to the reference sequences had a length between 28 and 33 nt (S2A Fig), within the range of previous observations [89–91]. Mapped RPFs of 28–32 nt long were selected to determine the codon occupancy. The ribosomal A sites where codon selection occurs were considered as nucleotide positions 16–18 and the 5' most nucleotide of each sequenced read was taken as position 1 [57]. After assigning the A site codons, the proportions of in-frame reads for the WT strain and Si*adat2* were 85.6% and 86.7%, respectively (S2B Fig). All out-of-frame reads were excluded from further analysis. To exclude the effect of the translation initiation and termination on ribosome occupancy, footprint reads mapping within the first and last 15 codons of coding sequences were excluded from our analyses. To compare different samples, normalization factors were introduced based on the total reads mapped to the reference, including RPFs, reads from rRNAs and tRNAs. The total reads were assumed to be equivalent for all samples. The transcript level of each gene was measured by RPKM, calculated as described previously [31]. To measure the relative codon occupancy of each codon in each codon family within A site, A+1 site, E site and P site, we used this method: RPFs at a given codon at a given position were normalized to the average per-codon read density in its open reading frame (ORF) [92]. To reflect the rank change of the relative codon occupancy and characterize their difference between the Si*adat2* and WT strains in each ADAT-related codon family, the relative codon occupancy of each codon was further transformed into a z-score using the formula z-score = (x-μ)/δ, where x is the codon occupancy of a codon, μ is the average of the codon occupancy of all codons in this codon family, and δ is the standard deviation of the relative codon occupancy of all codons in this codon family. This method can be used for comparison the relative codon occupancy between codons within each codon family, but it is affected by the average per-codon read density in ORFs. Because the average per-codon read density of most of the genes in the Si*adat2* is higher than that in the WT strain, which leads to systematically lower codon occupancy values in the Si*adat2*, therefore, it is not suitable for comparison of absolute codon occupancy between the WT strain and Si*adat2*. For the measurement of absolute codon occupancy, we used a previously reported method [31]. Briefly, the frequency of each codon within different sites was calculated and normalized to library size (total reads). The normalized frequency of each codon was further normalized to the mRNA level (RPKM). All quotients of each codon were summed and divided by the total number of the codon in this analysis, which is referred as the absolute codon occupancy for each sense codon. The analyses of ribosome profiling data were achieved through a collection of customized scripts written in Perl and R, which were uploaded to GitHub (https://github.com/lxlscc0715/scripts-for-ribosome-profiling-and-RNA-seq). Note that very similar results were obtained in the two independent biological replicates using these methods (S3A–S3F Fig).

To measure the ribosome density of all detected genes by ribosome profiling experiment, only the in-frame footprint reads were counted for each gene. The ribosome density of each gene was defined as the number of the mapped RPF reads per kilo base per million (RPKM of RPFs) divided by mRNA level (RPKM, obtained by the accompanying RNA-seq results).

### tRNA-seq and mRNA-seq

The tRNA-seq and mRNA-seq libraries were generated from the WT and Si*adat2* cultures with culture conditions identical to those described in the section of qRT-PCR experiment. To exclude the possible effect of amino acid starvation condition on tRNA I34 modification, the WT strain culture was treated with 5 mM 3-AT for 8 h before sample collection. Total RNAs were extracted using Trizol reagents (Invitrogen) and treated with DNase (Turbo DNase, Ambion). For tRNA-seq, the low molecular weight RNAs were enriched from total RNA samples as follows: An equal volume of total RNAs were mixed thoroughly with 10% PEG-8000 and 1 M NaCl and placed on ice for 30 min. After centrifugation at 14000g for 10 min, the supernatant was transferred in a new tube containing 3 volumes of 100% ethanol and 0.1 volume NaOAc. The samples were precipitated at -20°C for 2 h before centrifugation at 12000 rpm for 10 min. The pellets were washed twice using 70% ethanol and air dried before being dissolved in RNase-free water. tRNAs were further enriched by size selection (50–200 bp) in 12% TBE-urea gels (8 M urea, 12% 19:1 acrylamide/bisacrylamide, 1×TBE). The tRNAs were then de-aminoacylated in 50 mM Tris-HCl (pH 9.0) and incubated at 37°C for 30 min. The reaction was neutralized by the addition of an equal volume of 50 mM buffered acetate (pH 4.5) and 100 mM NaCl. tRNA libraries were prepared according to the protocol for ARTseq Ribosome Profiling Kit described previously [31]. Briefly, a 3’ adaptor was ligated into the tRNAs after end repair of the samples. RNAs were then reverse-transcribed at 50°C for 1 h (SuperScript III cDNA synthesis kit, Invitrogen). After cDNA size selection in 12% TBE-urea gels, the cDNAs were cyclized, followed by 12-cycle PCR amplification with Phusion DNA polymerase (Thermo Scientific). The PCR libraries were subjected to Illumina HiSeq2000 sequencing. The mRNA-seq was performed by Joint Genome Institute (JGI). The tRNA-seq and mRNA-seq reads that mapped uniquely to the reference with at most two mismatches were considered. The tRNA and mRNA references were from GtRNAdb(http://gtrnadb.ucsc.edu/GtRNAdb2/genomes/eukaryota/Neur_cras_OR74A/) and GenBank (https://www.ncbi.nlm.nih.gov/bioproject/13841), respectively.

The raw and processed sequencing data from this study have been submitted to the NCBI Gene Expression Omnibus under accession number GSE130155.

### Gene functional enrichment analysis

The functional category enrichment (including Gene Ontology (GO), Interpro, and KEGG terms) was analyzed with the functional annotation tool of the DAVID bioinformatics web server (http://david.abcc.ncifcrf.gov/). For each enrichment analysis, the up-regulated or down-regulated genes in the Si*adat2* strain compared with the WT strain, at the ribosome density level, protein level or mRNA level, were used as the targeted gene lists, and the whole genome annotation was used as background. The enriched genes of each functional category, the enrichment fold, and the various statistical parameters of the enrichment analysis including p-values, Bonferroni-corrected p-values, Benjamini-corrected p-values, and FDR values are listed in Supplementary S1 Table. The functional categories shown in figures in the main text come from KEGG database annotation.

## Supporting information

S1 Fig tRNA-seq experiments for wild-type *Neurospora* samples under different growth conditions. **(A)** Samples for tRNA-seq were from mycelia dics cultured in liquid rich medium (2% glucose medium), aerial hyphae and conidia cultured in solid minimal medium, respectively. **(B)** The results of an independent biological replicate of the tRNA-seq for the WT and Si*adat2* strains and the WT strain treated with 3-AT (WT+3AT), corresponding to Fig 2C. Bar charts showing the proportions of G, A, C, and U contents at the position 34 of each tRNA detected by tRNA sequencing. Inosine was read as G by sequencing. The tRNA species with anticodons are indicated at the top of each bar chart.
(PDF)

S2 Fig Ribosome profiling data analysis. **(A)** The length distributions of all mapped reads from the WT and Si*adat2* strains. **(B)** The proportions of in-frame reads and out-of-frame reads from the WT and Si*adat2* strains. **(C)** Average coverage of 5’ nucleotides from ribosome footprint reads mapping near start codons (left) and stop codons (right) across all transcripts in the WT (upper) and Si*adat2* (lower) strains. Clear periodicity was seen in both samples.
(PDF)

S3 Fig The reproducibility of ribosome profiling experiment in this study. **(A, B)** Correlation of the relative codon occupancy of the WT strain (A) and the Si*adat2* (B) between two independent biological replicates. **(C)** Correlation of the relative codon occupancy change fold of the WT strain and Si*adat2* between two independent biological replicates. **(D, E)** Correlation of the absolute codon occupancy of the WT strain (D) and the Si*adat2* (E) between two independent biological replicates. **(F)** Correlation of the absolute codon occupancy change fold of the WT strain and Si*adat2* between two independent biological replicates.
(PDF)

S4 Fig Comparison of the relative codon occupancy within A, A+1, P and E sites, related to Fig 4A and 4B. **(A)** Comparison of the relative codon occupancies in each ADAT-related codon family between the WT and Si*adat2* strains. Red and blue indicate the ADAT-related NNC and NNU codons, respectively. **(B)** The relative codon occupancies of the eight ADAT-related codons in each family within A+1, P and E sites. The relative codon occupancy values in each codon family were normalized and centralized by z-score transformation. The averages of the relative codon occupancies from two independent biological replicates for the WT and Si*adat2* strains, respectively, are shown in A and B.
(PDF)

S5 Fig The CHX treatment of cultures before sample collection and the glucose concentration in medium had little effect on the relative codon occupancy in *N*. *crassa*. **(A, B)** Correlation of the relative codon occupancy of 61 codons between samples with/without CHX treatment (all samples were cultured in 0.1% glucose medium). **(C)** Comparison of the relative codon occupancy in the eight ADAT-related codon families from cultures with/without CHX treatment (cultured in 0.1% glucose medium or 2% glucose medium as indicated).
(PDF)

S6 Fig Ribosome profiling results demonstrated that limited carbon source and amino acid starvation did not alter the relative codon occupancy of ADAT-related NNC and NNU codons *in vivo*. The relative codon occupancy values in each ADAT-related codon family were normalized and centralized by z-score transformation. Cultures were grown in rich carbon source medium (2% glucose medium), low carbon source medium (lowC, 0.1% glucose medium), low carbon and no nitrogen source medium (lowC & noN, 0.1% glucose medium without nitrogen source) and lys4 deletion mutant (Δlys4) cultured in 2% glucose medium with 2 mg/mL lysine, respectively.
(PDF)

S7 Fig The effect of *adat2* silencing on codon occupancy fold changes of non-ADAT-related codons in the Si*adat2* compared with that in the WT strain, related to Fig 4C. Genome-wide codon usage frequency (numbers per thousand codons, upper panel) in *N*. *crassa* and codon occupancy change folds (lower panel) in non-ADAT-related codon families between the Si*adat2* and WT cells. Data from two independent biological replicates are shown. The codon occupancy values are normalized to that of the most occupied codon (5’-CGA-3’, arginine).
(PDF)

S8 Fig A scattered plot showing the ribosome density of each gene in the WT and Si*adat2* strains in the second independent biological replicated experiment, related to Fig 5A. The genes with up-regulated, down-regulated, and unchanged ribosome density in the Si*adat2* compared to the WT strain are indicated by green, blue, and yellow dots, respectively. RPGs are marked as red dots.
(PDF)

S9 Fig The reproducibility of quantitative MS and mRNA-seq experiments in this study. **(A)** The correlation of protein level fold change (Si*adat2*/WT) in two independent biological replicates, detected by quantitative MS analysis. **(B)** The correlation of mRNA levels in the WT strain in two independent biological replicates. **(C)** The correlation of mRNA levels in the Si*adat2* in two independent biological replicates.
(PDF)

S10 Fig Ribosome occupancy of *GCN4* transcript in yeast from two previous studies. A schematic of the *GCN4* transcript is shown at the top. The histograms in red box represent the normalized number of RPFs on each codon of *GCN4* transcript in the BY4741 (background strain) and *kti11*Δ strains [78]. The histograms in blue box represent normalized number of the RPFs on each codon of the *GCN4* transcript in yCW30 with/without 3-AT treatment (Guydosh & Green, 2014).
(PDF)

S11 Fig Multiple sequence alignments of the coding sequences of the WT and the codon optimized or de-optimized versions of luciferase. * indicates conserved sites.
(PDF)

S1 Table Gene functional enrichment analyses based on ribosome density, protein level and mRNA level differences in the Si*adat2* compared to the WT strain. (XLSX)

S2 Table The results of qualitative MS experiments. (XLSX)

S3 Table The results of mRNA-seq experiments. (XLSX)
