# Supplementary figures and images for: Adaptation of codon usage to tRNA I34 modification controls translation kinetics and proteome landscape
Source: PLoS Genet. 2020 Jun 1;16(6):e1008836. doi: 10.1371/journal.pgen.1008836 (PMC7289440; doi:10.1371/journal.pgen.1008836)

S1 Fig

A

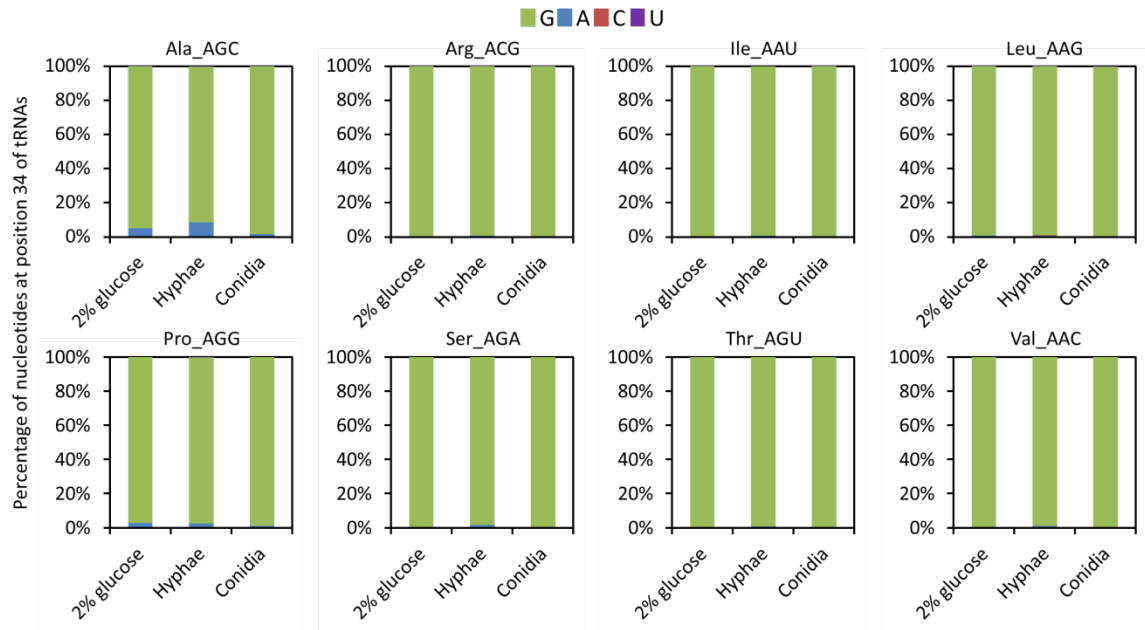

B

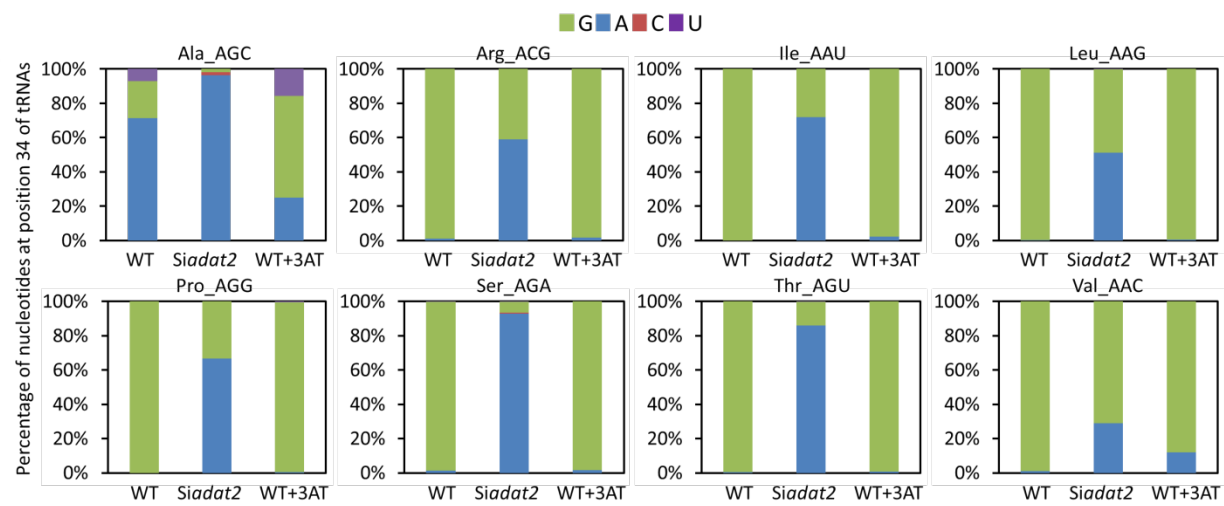

Supplement: S1 Fig — (A) Samples for tRNA-seq were from mycelia dics cultured in liquid rich medium (2% glucose medium), aerial hyphae and conidia cultured in solid minimal medium, respectively. (B) The results of an independent biological replicate of the tRNA-seq for the WT and Siadat2 strains and the WT strain treated with 3-AT (WT+3AT), corresponding to Fig 2C. Bar charts showing the proportions of G, A, C, and U contents at the position 34 of each tRNA detected by tRNA sequencing. Inosine was read as G by sequencing. The tRNA species with anticodons are indicated at the top of each bar chart. (PDF) [file pgen.1008836.s001.pdf]

S2 Fig

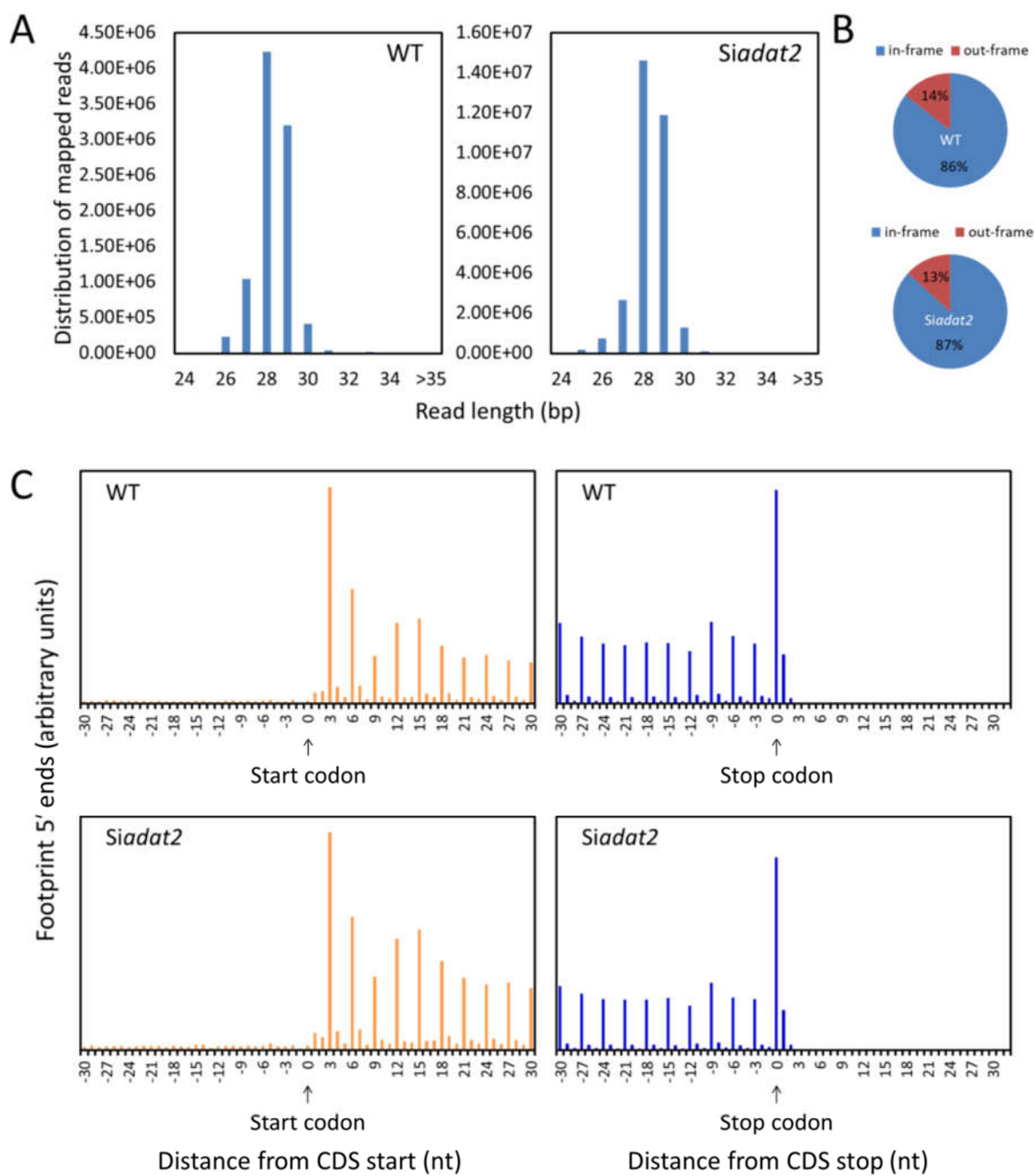

Supplement: S2 Fig — (A) The length distributions of all mapped reads from the WT and Siadat2 strains. (B) The proportions of in-frame reads and out-of-frame reads from the WT and Siadat2 strains. (C) Average coverage of 5’ nucleotides from ribosome footprint reads mapping near start codons (left) and stop codons (right) across all transcripts in the WT (upper) and Siadat2 (lower) strains. Clear periodicity was seen in both samples. (PDF) [file pgen.1008836.s002.pdf]

S3 Fig

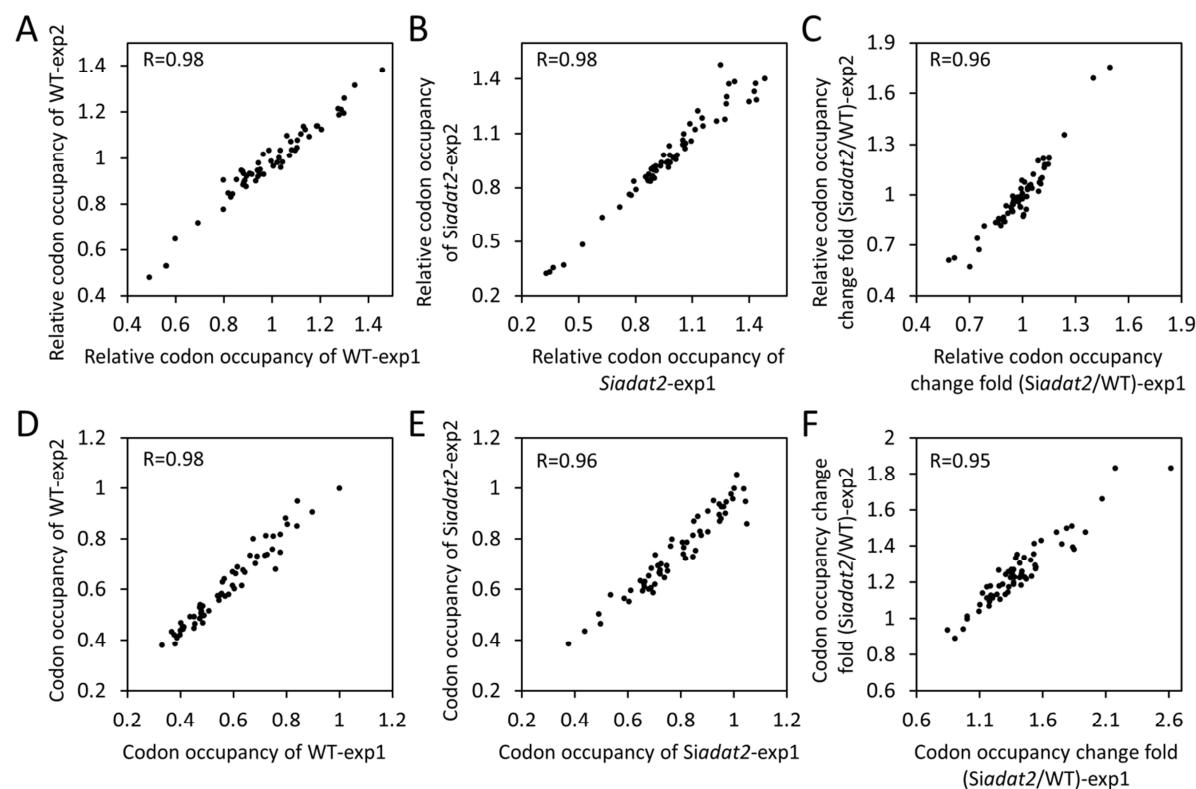

Supplement: S3 Fig — (A, B) Correlation of the relative codon occupancy of the WT strain (A) and the Siadat2 (B) between two independent biological replicates. (C) Correlation of the relative codon occupancy change fold of the WT strain and Siadat2 between two independent biological replicates. (D, E) Correlation of the absolute codon occupancy of the WT strain (D) and the Siadat2 (E) between two independent biological replicates. (F) Correlation of the absolute codon occupancy change fold of the WT strain and Siadat2 between two independent biological replicates. (PDF) [file pgen.1008836.s003.pdf]

S4 Fig

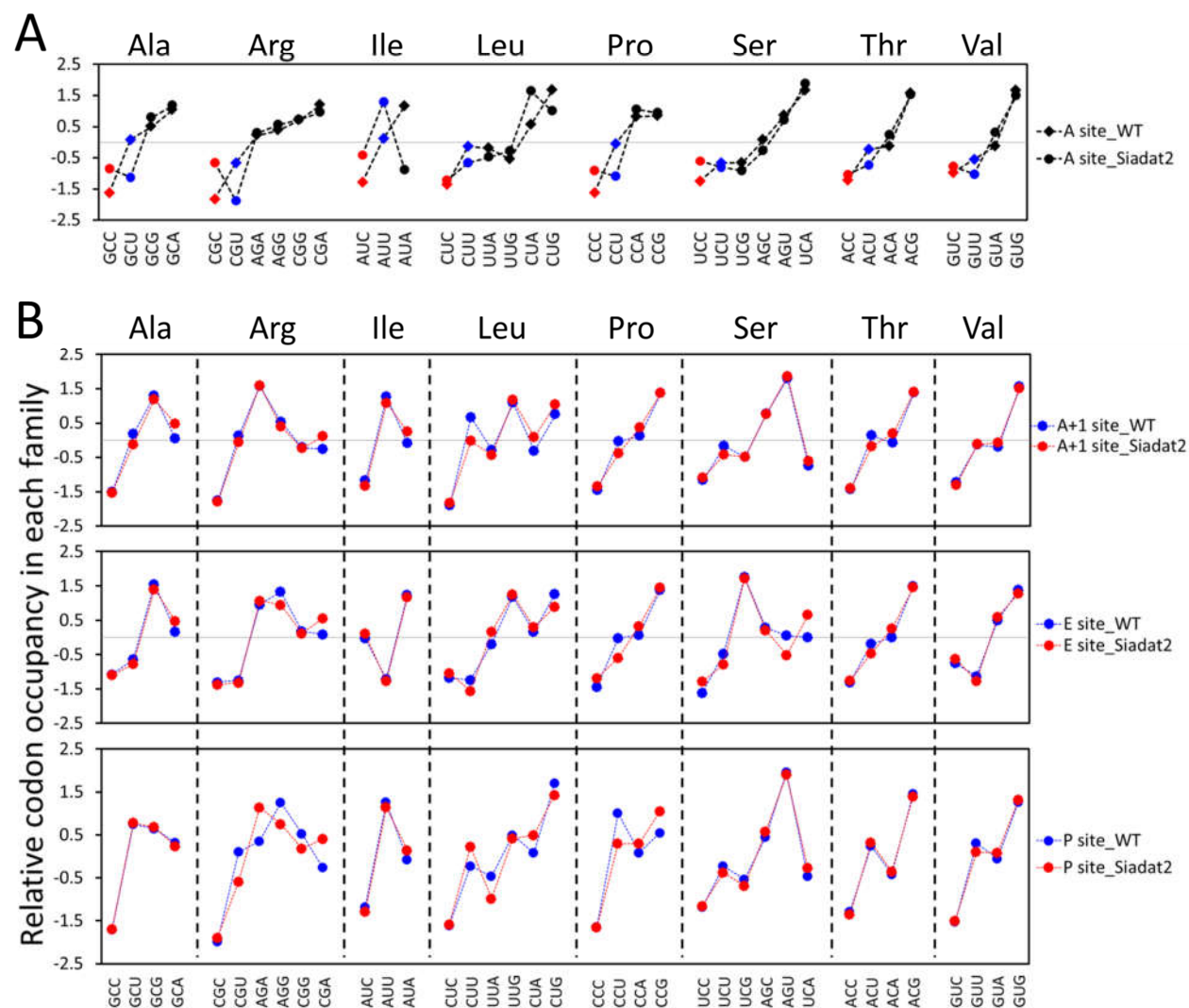

Supplement: S4 Fig — (A) Comparison of the relative codon occupancies in each ADAT-related codon family between the WT and Siadat2 strains. Red and blue indicate the ADAT-related NNC and NNU codons, respectively. (B) The relative codon occupancies of the eight ADAT-related codons in each family within A+1, P and E sites. The relative codon occupancy values in each codon family were normalized and centralized by z-score transformation. The averages of the relative codon occupancies from two independent biological replicates for the WT and Siadat2 strains, respectively, are shown in A and B. (PDF) [file pgen.1008836.s004.pdf]

S5 Fig

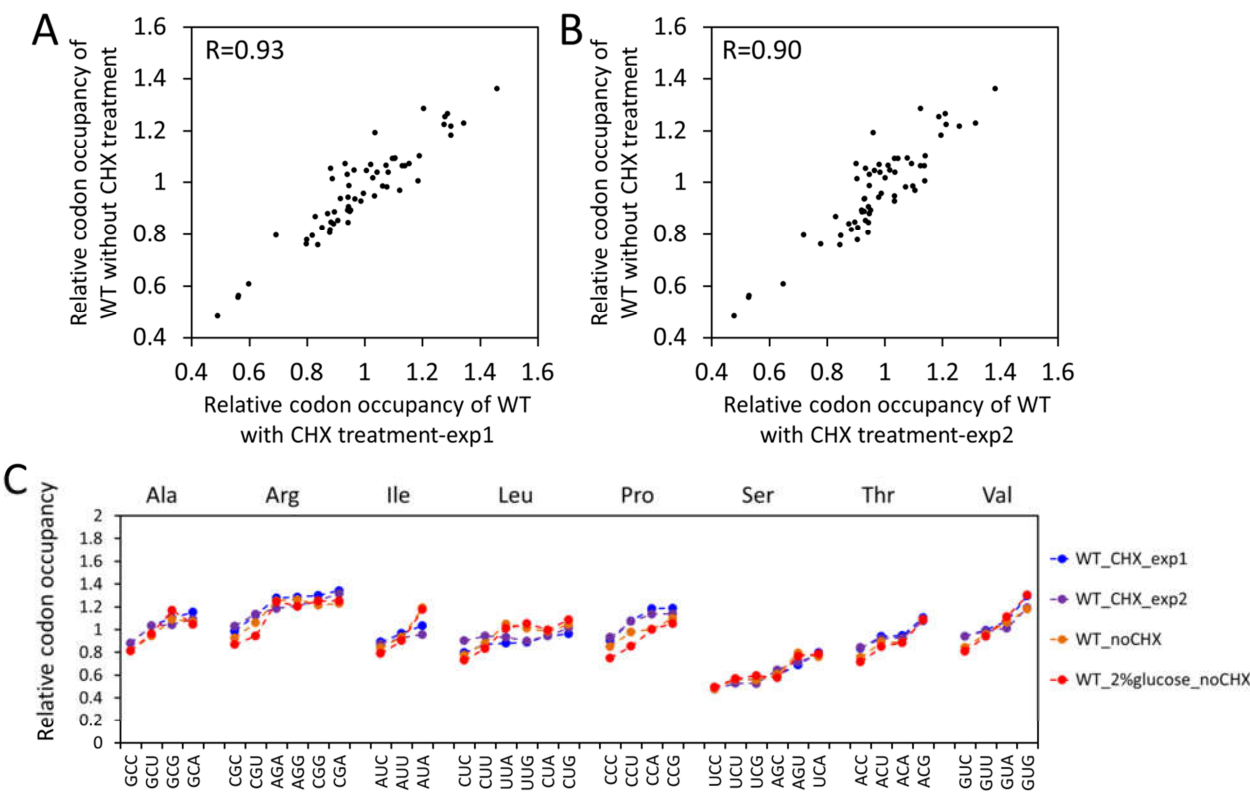

Supplement: S5 Fig — (A, B) Correlation of the relative codon occupancy of 61 codons between samples with/without CHX treatment (all samples were cultured in 0.1% glucose medium). (C) Comparison of the relative codon occupancy in the eight ADAT-related codon families from cultures with/without CHX treatment (cultured in 0.1% glucose medium or 2% glucose medium as indicated). (PDF) [file pgen.1008836.s005.pdf]

S6 Fig

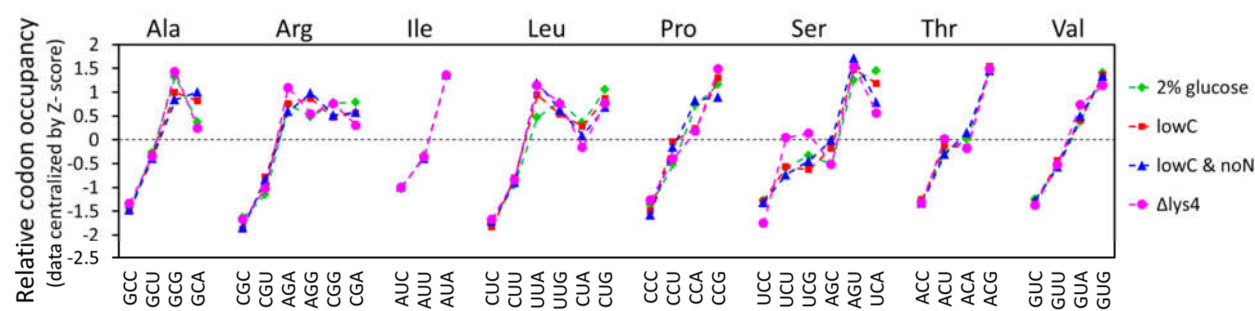

Supplement: S6 Fig — The relative codon occupancy values in each ADAT-related codon family were normalized and centralized by z-score transformation. Cultures were grown in rich carbon source medium (2% glucose medium), low carbon source medium (lowC, 0.1% glucose medium), low carbon and no nitrogen source medium (lowC & noN, 0.1% glucose medium without nitrogen source) and lys4 deletion mutant (Δlys4) cultured in 2% glucose medium with 2 mg/mL lysine, respectively. (PDF) [file pgen.1008836.s006.pdf]

S7 Fig

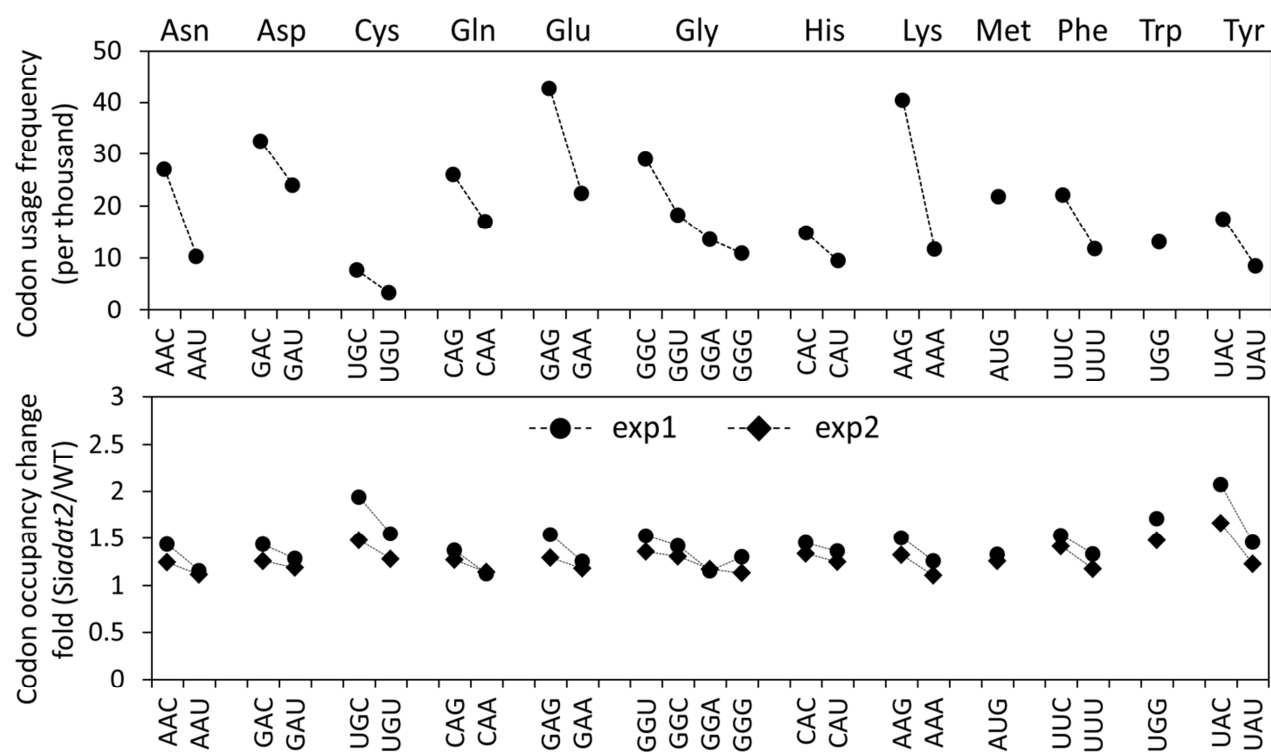

Supplement: S7 Fig — Genome-wide codon usage frequency (numbers per thousand codons, upper panel) in N. crassa and codon occupancy change folds (lower panel) in non-ADAT-related codon families between the Siadat2 and WT cells. Data from two independent biological replicates are shown. The codon occupancy values are normalized to that of the most occupied codon (5’-CGA-3’, arginine). (PDF) [file pgen.1008836.s007.pdf]

S8 Fig

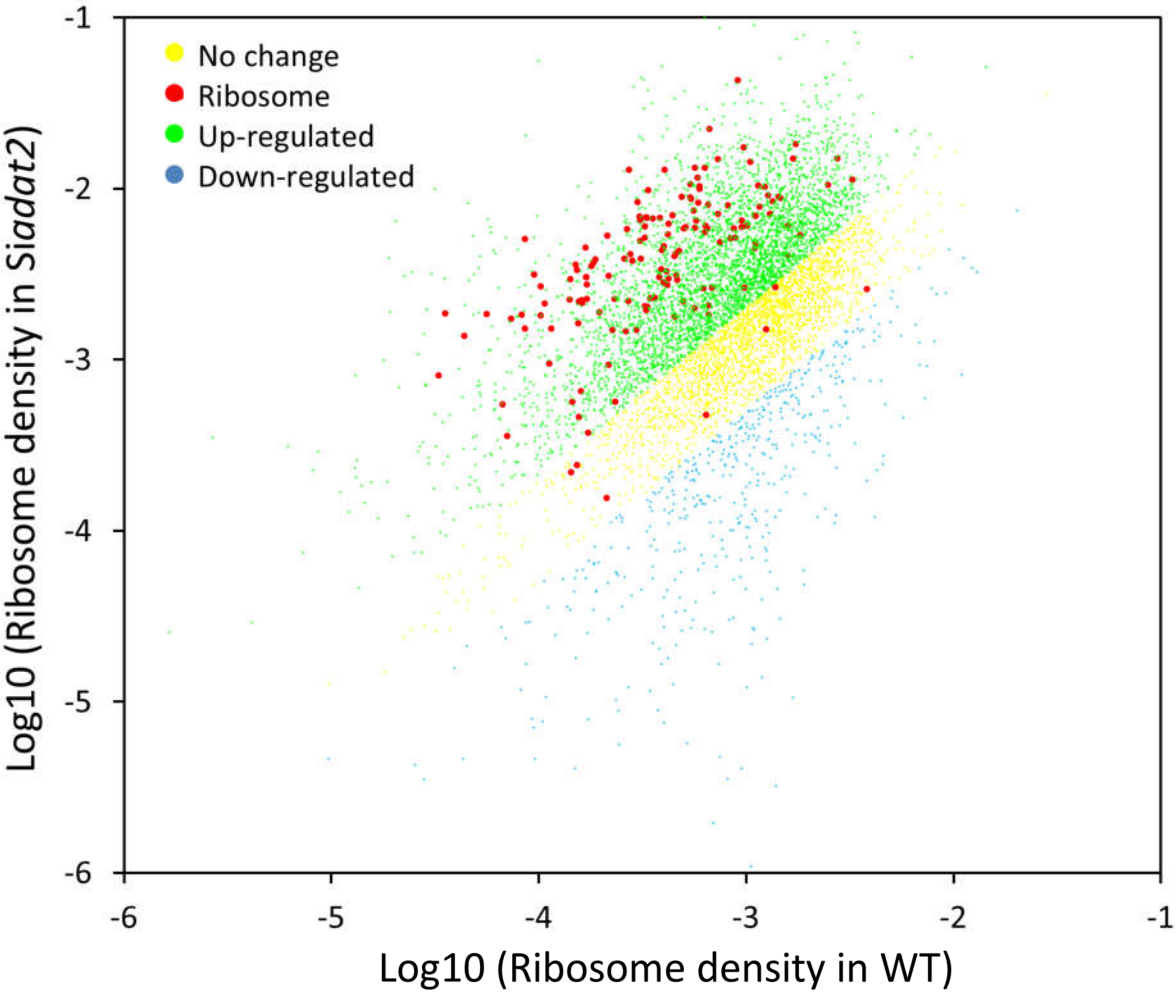

Supplement: S8 Fig — The genes with up-regulated, down-regulated, and unchanged ribosome density in the Siadat2 compared to the WT strain are indicated by green, blue, and yellow dots, respectively. RPGs are marked as red dots. (PDF) [file pgen.1008836.s008.pdf]

S9 Fig

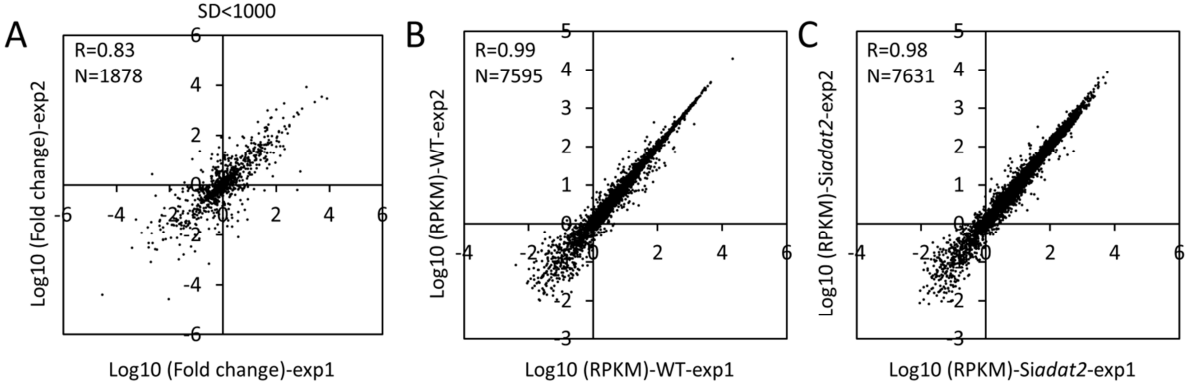

Supplement: S9 Fig — (A) The correlation of protein level fold change (Siadat2/WT) in two independent biological replicates, detected by quantitative MS analysis. (B) The correlation of mRNA levels in the WT strain in two independent biological replicates. (C) The correlation of mRNA levels in the Siadat2 in two independent biological replicates. (PDF) [file pgen.1008836.s009.pdf]

S10 Fig

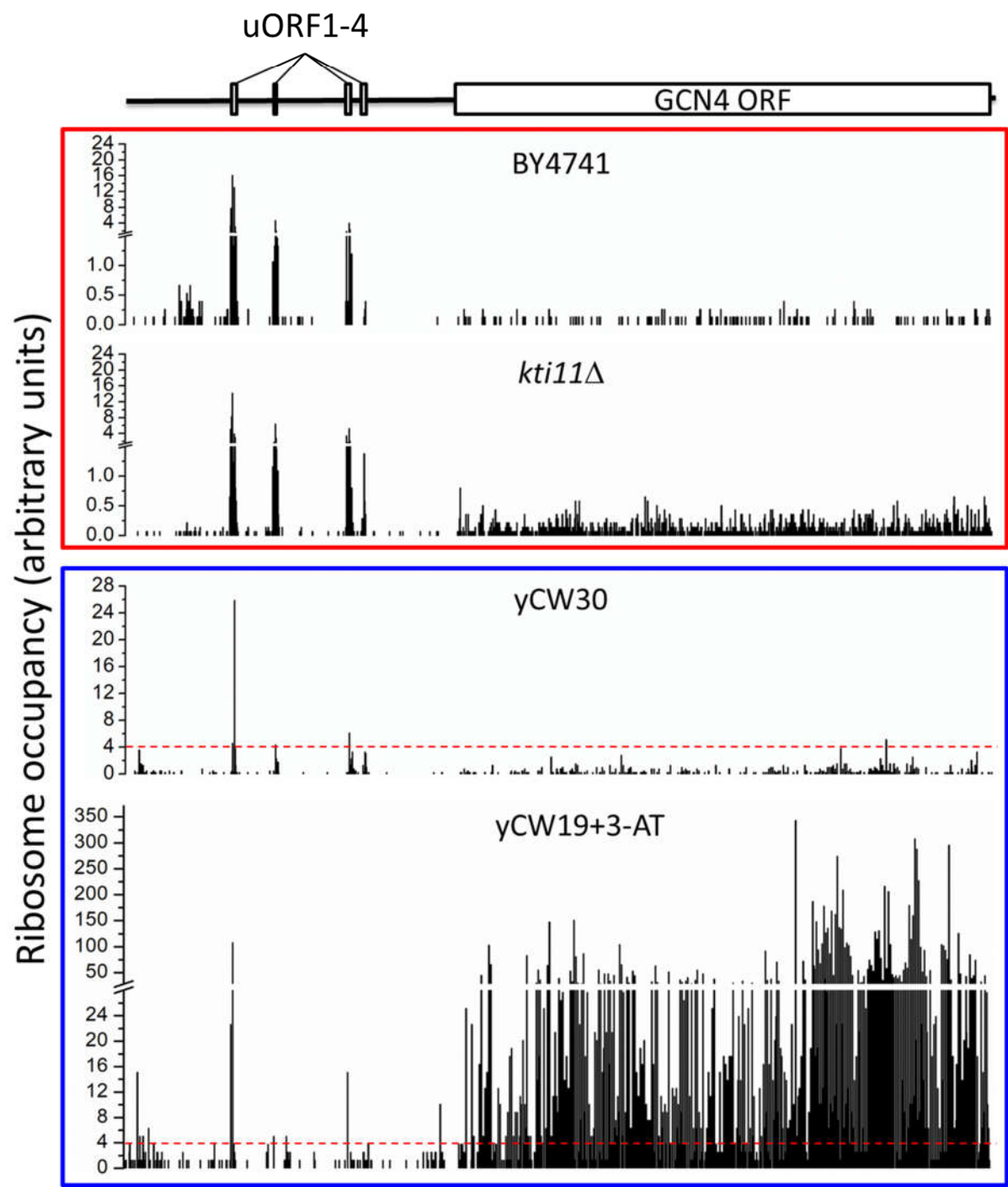

Supplement: S10 Fig — A schematic of the GCN4 transcript is shown at the top. The histograms in red box represent the normalized number of RPFs on each codon of GCN4 transcript in the BY4741 (background strain) and kti11Δ strains [78]. The histograms in blue box represent normalized number of the RPFs on each codon of the GCN4 transcript in yCW30 with/without 3-AT treatment (Guydosh & Green, 2014). (PDF) [file pgen.1008836.s010.pdf]
