# Supplementary material for: Adaptation of codon usage to tRNA I34 modification controls translation kinetics and proteome landscape
Source: PLoS Genet. 2020 Jun 1;16(6):e1008836. doi: 10.1371/journal.pgen.1008836 (PMC7289440; doi:10.1371/journal.pgen.1008836)
Supplement: S11 Fig — * indicates conserved sites. (PDF) [file pgen.1008836.s011.pdf]

S11 Fig

For luciferase gene coding sequence:

```
OPT(C→U)-luc    ATGGAGGACGCCAAGAACATTAAGAAGGGCCCCGCTCCCTTCTACCCTCTCGAGGACGGCACTGCCGGCGAGCAGCTTCACAAGGCCATGAAGCGTTACGCCCTTGTCCCTGGCACTATT
OPT-luc         ATGGAGGACGCCAAGAACATCAAGAAGGGCCCCGCCCCCTTCTACCCCCTCGAGGACGGCACCGCCGGCGAGCAGCTCCACAAGGCCATGAAGCGCTACGCCCTCGTCCCCGGCACCATC
WT-luc          ATGGAGGACGCCAAAAACATAAAGAAAGGCCCGGCGCCATTCTATCCGCTGGAAGATGGAACCGCTGGAGAGCAACTGCATAAGGCTATGAAGAGATACGCCCTGGTTCCTGGAACAATT
                ************** ***** ***** ***** ** ** ***** ** ** ** ** ** ** ** ** ***** ** ** ***** ****** * ******** ** ** ** ** **

OPT(C→U)-luc    GCCTTCACTGACGCCCACATTGAGGTCGACATTACTTACGCTGAGTACTTCGAGATGTCTGTTCGCCTTGCCGAGGCTATGAAGCGCTACGGCCTTAACACTAACCACCGTATCGTTGTC
OPT-luc         GCCTTCACCGACGCCCACATCGAGGTCGACATCACCTACGCCGAGTACTTCGAGATGTCCGTCCGCCTCGCCGAGGCCATGAAGCGCTACGGCCTCAACACCAACCACCGCATCGTCGTC
WT-luc          GCTTTTACAGATGCACATATCGAGGTGGACATCACTTACGCTGAGTACTTCGAAATGTCCGTTCGGTTGGCAGAAGCTATGAAACGATATGGGCTGAATACAAATCACAGAATCGTCGTA
                ** ** ** ** ** ** ** ***** ***** ** ***** *********** ***** ** **  * ** ** ** ***** ** ** ** ** ** ** ** *** * ***** **

OPT(C→U)-luc    TGCTCTGAGAACTCTCTTCAGTTCTTCATGCCTGTTCTCGGCGCTCTCTTCATTGGCGTCGCTGTCGCTCCCGCTAACGACATCTACAACGAGCGTGAGCTCCTTAACTCTATGGGCATT
OPT-luc         TGCTCCGAGAACTCCCTCCAGTTCTTCATGCCCGTCCTCGGCGCCCTCTTCATCGGCGTCGCCGTCGCCCCCGCCAACGACATCTACAACGAGCGCGAGCTCCTCAACTCCATGGGCATC
WT-luc          TGCAGTGAAAACTCTCTTCAATTCTTTATGCCGGTGTTGGGCGCGTTATTTATCGGAGTTGCAGTTGCGCCCGCGAACGACATTTATAATGAACGTGAATTGCTCAACAGTATGGGCATT
                ***    ** ***** ** ** ***** ***** **  * *****  * ** ** ** ** ** ** ** ***** ******** ** ** ** ** **  * ** ***    ********

OPT(C→U)-luc    TCTCAGCCTACTGTTGTCTTCGTTTCCAAGAAGGGCCTTCAGAAGATCCTTAACGTCCAGAAGAAGCTTCCTATTATCCAGAAGATTATCATTATGGACTCTAAGACTGACTACCAGGGC
OPT-luc         TCCCAGCCCACCGTCGTCTTCGTCTCCAAGAAGGGCCTCCAGAAGATCCTCAACGTCCAGAAGAAGCTCCCCATCATCCAGAAGATCATCATCATGGACTCCAAGACCGACTACCAGGGC
WT-luc          TCGCAGCCTACCGTGGTGTTCGTTTCCAAAAAGGGGTTGCAAAAAATTTTGAACGTGCAAAAAAAGCTCCCAATCATCCAAAAAATTATTATCATGGATTCTAAAACGGATTACCAGGGA
                ** ***** ** ** ** ***** ***** *****  * ** ** **  * ***** ** ** ***** ** ** ***** ** ** ** ** ***** ** ** ** ** ********

OPT(C→U)-luc    TTCCAGTCTATGTACACTTTCGTCACTTCTCACCTTCCCCCTGGCTTCAACGAGTACGACTTCGTCCCTGAGTCCTTCGACCGTGACAAGACTATTGCCCTTATCATGAACTCTTCTGGC
OPT-luc         TTCCAGTCCATGTACACCTTCGTCACCTCCCACCTCCCCCCCGGCTTCAACGAGTACGACTTCGTCCCCGAGTCCTTCGACCGCGACAAGACCATCGCCCTCATCATGAACTCCTCCGGC
WT-luc          TTTCAGTCGATGTACACGTTCGTCACATCTCATCTACCTCCCGGTTTTAATGAATACGATTTTGTGCCAGAGTCCTTCGATAGGGACAAGACAATTGCACTGATCATGAACTCCTCTGGA
                ** ***** ******** ******** ** ** ** ** ** ** ** ** ** ***** ** ** ** ***********  * ******** ** ** ** *********** ** **

OPT(C→U)-luc    TCTACCGGCCTTCCCAAGGGCGTTGCCCTTCCTCACCGTACCGCTTGCGTCCGTTTCTCCCACGCTCGCGACCCTATCTTCGGCAACCAGATTATCCCTGACACTGCTATCCTTTCTGTT
OPT-luc         TCCACCGGCCTCCCCAAGGGCGTCGCCCTCCCCCACCGCACCGCCTGCGTCCGCTTCTCCCACGCCCGCGACCCCATCTTCGGCAACCAGATCATCCCCGACACCGCCATCCTCTCCGTC
WT-luc          TCTACTGGTCTGCCTAAAGGTGTCGCTCTGCCTCATAGAACTGCCTGCGTGAGATTCTCGCATGCCAGAGATCCTATTTTTGGCAATCAAATCATTCCGGATACTGCGATTTTAAGTGTT
                ** ** ** ** ** ** ** ** ** ** ** **  * ** ** *****  * ***** ** **  * ** ** ** ** ***** ** ** ** ** ** ** ** **  *    **

OPT(C→U)-luc    GTCCCTTTCCACCACGGCTTCGGCATGTTCACTACTCTCGGCTACCTTATCTGCGGCTTCCGTGTCGTTCTCATGTACCGTTTCGAGGAGGAGCTCTTCCTTCGCTCTCTCCAGGACTAC
OPT-luc         GTCCCCTTCCACCACGGCTTCGGCATGTTCACCACCCTCGGCTACCTCATCTGCGGCTTCCGCGTCGTCCTCATGTACCGCTTCGAGGAGGAGCTCTTCCTCCGCTCCCTCCAGGACTAC
WT-luc          GTTCCATTCCATCACGGTTTTGGAATGTTTACTACACTCGGATATTTGATATGTGGATTTCGAGTCGTCTTAATGTATAGATTTGAAGAAGAGCTGTTTCTGAGGAGCCTTCAGGATTAC
                ** ** ***** ***** ** ** ***** ** ** ***** **  * ** ** ** ** ** *****  * *****  * ** ** ** ***** ** **  *    ** ***** ***

OPT(C→U)-luc    AAGATTCAGTCCGCTCTCCTTGTCCCTACCCTTTTCTCTTTCTTCGCTAAGTCTACTCTCATTGACAAGTACGACCTCTCTAACCTCCACGAGATTGCCTCTGGCGGCGCCCCTCTCTCT
OPT-luc         AAGATCCAGTCCGCCCTCCTCGTCCCCACCCTCTTCTCCTTCTTCGCCAAGTCCACCCTCATCGACAAGTACGACCTCTCCAACCTCCACGAGATCGCCTCCGGCGGCGCCCCCCTCTCC
WT-luc          AAGATTCAAAGTGCGCTGCTGGTGCCAACCCTATTCTCCTTCTTCGCCAAAAGCACTCTGATTGACAAATACGATTTATCTAATTTACACGAAATTGCTTCTGGTGGCGCTCCCCTCTCT
                ***** **    ** ** ** ** ** ***** ***** ******** **    ** ** ** ***** *****  * ** **  * ***** ** ** ** ** ***** ** *****

OPT(C→U)-luc    AAGGAGGTCGGCGAGGCTGTCGCTAAGCGCTTCCACCTTCCCGGCATTCGCCAGGGCTACGGCCTTACTGAGACTACTTCTGCCATTCTCATTACTCCTGAGGGCGACGACAAGCCTGGC
OPT-luc         AAGGAGGTCGGCGAGGCCGTCGCCAAGCGCTTCCACCTCCCCGGCATCCGCCAGGGCTACGGCCTCACCGAGACCACCTCCGCCATCCTCATCACCCCCGAGGGCGACGACAAGCCCGGC
WT-luc          AAGGAAGTCGGGGAAGCGGTTGCCAAGAGGTTCCATCTGCCAGGTATCAGGCAAGGATATGGGCTCACTGAGACTACATCAGCTATTCTGATTACACCCGAGGGGGATGATAAACCGGGC
                ***** ***** ** ** ** ** *** * ***** ** ** ** **  * ** ** ** ** ** ***** ** ** ** ** ** ** ** ** ***** ** ** ** ** ***
```

```
OPT(C→U)-luc   GCTGTCGGCAAGGTTGTCCCTTTCTTCGAGGCCAAGGTTGTCGACCTTGACACCGGCAAGACTCTCGGCGTTAACCAGCGCGGCGAGCTTTGCGTCCGTGGCCCCATGATTATGTCTGGC
OPT-luc        GCCGTCGGCAAGGTCGTCCCCTTCTTCGAGGCCAAGGTCGTCGACCTCGACACCGGCAAGACCCTCGGCGTCAACCAGCGCGGCGAGCTCTGCGTCCGCGGCCCCATGATCATGTCCGGC
WT-luc         GCGGTCGGTAAAGTTGTTCCATTTTTTGAAGCGAAGGTTGTGGATCTGGATACCGGGAAAACGCTGGGCGTTAATCAAAGAGGCGAACTGTGTGTGAGAGGTCCTATGATTATGTCCGGT
               ** ***** ** ** ** ** ** ** ** ** ***** ** ** ** ** ***** ** ** ** ***** ** **   * ***** ** ** **   * ** ** ***** ***** **

OPT(C→U)-luc   TACGTTAACAACCCTGAGGCTACTAACGCTCTCATTGACAAGGACGGCTGGCTCCACTCTGGCGACATCGCTTACTGGGACGAGGACGAGCACTTCTTCATCGTTGACCGCCTTAAGTCC
OPT-luc        TACGTCAACAACCCCGAGGCCACCAACGCCCTCATCGACAAGGACGGCTGGCTCCACTCCGGCGACATCGCCTACTGGGACGAGGACGAGCACTTCTTCATCGTCGACCGCCTCAAGTCC
WT-luc         TATGTAAACAATCCGGAAGCGACCAACGCCTTGATTGACAAGGATGGATGGCTACATTCTGGAGACATAGCTTACTGGGACGAAGACGAACACTTCTTCATCGTTGACCGCCTGAAGTCT
               ** ** ***** ** ** ** ** *****   * ** ******** ** ***** ** ** ** ***** ** *********** ***** ************** ******** *****

OPT(C→U)-luc   CTTATCAAGTACAAGGGCTACCAGGTTGCCCCTGCCGAGCTTGAGTCTATTCTCCTTCAGCACCCTAACATTTTCGACGCCGGCGTTGCCGGCCTTCCCGACGACGACGCTGGCGAGCTC
OPT-luc        CTCATCAAGTACAAGGGCTACCAGGTCGCCCCCGCCGAGCTCGAGTCCATCCTCCTCCAGCACCCCAACATCTTCGACGCCGGCGTCGCCGGCCTCCCCGACGACGACGCCGGCGAGCTC
WT-luc         CTGATTAAGTACAAAGGCTATCAGGTGGCTCCCGCTGAATTGGAATCCATCTTGCTCCAACACCCCAACATCTTCGACGCAGGTGTCGCAGGTCTTCCCGACGATGACGCCGGTGAACTT
               ** ** ******** ***** ***** ** ** ** **   * ** ** **   * ** ** ***** ***** ******** ** ** ** ** ** ******** ***** ** ** **

OPT(C→U)-luc   CCTGCCGCTGTCGTTGTCCTTGAGCACGGCAAGACTATGACTGAGAAGGAGATCGTTGACTACGTCGCTTCCCAGGTTACTACTGCCAAGAAGCTTCGCGGCGGCGTTGTCTTCGTTGAC
OPT-luc        CCCGCCGCCGTCGTCGTCCTCGAGCACGGCAAGACCATGACCGAGAAGGAGATCGTCGACTACGTCGCCTCCCAGGTCACCACCGCCAAGAAGCTCCGCGGCGGCGTCGTCTTCGTCGAC
WT-luc         CCCGCCGCCGTTGTTGTTTTGGAGCACGGAAAGACGATGACGGAAAAAGAGATCGTGGATTACGTCGCCAGTCAAGTAACAACCGCGAAAAAGTTGCGCGGAGGAGTTGTGTTTGTGGAC
               ** ***** ** ** **   * ******** ***** ***** ** ** ******** ** ********     ** ** ** ** ** ** *** * ***** ** ** ** ** ** ***

OPT(C→U)-luc   GAGGTCCCTAAGGGCCTTACCGGCAAGCTTGACGCCCGTAAGATCCGTGAGATCCTTATCAAGGCTAAGAAGGGCGGCAAGATCGCCGTCTGA
OPT-luc        GAGGTCCCCAAGGGCCTCACCGGCAAGCTCGACGCCCGCAAGATCCGCGAGATCCTCATCAAGGCCAAGAAGGGCGGCAAGATCGCCGTCTGA
WT-luc         GAAGTACCGAAAGGTCTTACCGGAAAACTCGACGCAAGAAAAATCAGAGAGATCCTCATAAAGGCCAAGAAGGGCGGAAAGATCGCCGTGTAA
               ** ** ** ** ** ** ***** ** ** *****   * ** *** * ******** ** ***** *********** *********** * *
```
